# Risk factors for catheter-related thrombosis in cancer patients: a systematic review

**DOI:** 10.3389/fcvm.2026.1914685

**Published:** 2026-09-18

**Authors:** Yilin Xie, Xueling Huang, Jiaying Zheng, Zhiyu Liang, Jiajia Huang, Youwei Su, Yunfeng Jiang, Pengpeng Wang

**Affiliations:** Nursing College of Guangxi Medical University, Nanning, Guangxi, China

**Keywords:** cancer, central venous catheters, risk factors, thrombosis, vascular access devices

## Abstract

**Background:**

Central venous access devices (CVADs) are indispensable in cancer care, particularly for chemotherapy administration, nutritional support, and blood product transfusion. Catheter-related thrombosis (CRT), one of the most common non-infectious complications associated with CVADs, may result in treatment delays, increased healthcare costs, and potentially life-threatening events such as pulmonary embolism. Although numerous studies have investigated risk factors for CRT among cancer patients, no comprehensive systematic review has synthesized existing evidence, which hinders the formulation of evidence-based prevention and intervention strategies.

**Methods:**

Comprehensive literature searches were performed in PubMed, Embase, Web of Science and the Cochrane Library from database establishment to February 2026. Two researchers independently screened eligible studies and extracted data based on pre-defined inclusion and exclusion criteria; any discrepancies were resolved via discussion with a third researcher.

**Results:**

A total of 58 studies covering 50,987 cancer patients were included in this review. Risk factors for CRT in cancer patients were categorized into three major groups: patient-related factors, catheter-related factors, and treatment-related factors.

**Conclusion:**

This systematic review synthesised risk factors for CRT in cancer patients and classified them into three dimensions concerning patients, catheters and treatments. The findings can provide evidence for clinicians to formulate targeted prevention measures and optimize catheter management protocols.

## 1 Introduction

Central venous access devices (CVADs), including peripherally inserted central catheters (PICCs), implantable ports, and tunnelled central venous catheters, have become indispensable in cancer care and are widely used for chemotherapy administration, parenteral nutritional support, long-term intravenous therapy, and blood product transfusion. Catheter-related thrombosis (CRT) refers to venous thrombus formation caused by vascular endothelial injury, abnormal blood flow, or a hypercoagulable state resulting from the placement of CVADs. It is one of the most common non-infectious complications associated with CVADs (1).

It has been reported that the incidence of CRT in cancer patients varies considerably, with asymptomatic thrombosis ranging from 8.8% to 18% and symptomatic thrombosis ranging from 2.7% to 8%. CRT not only leads to catheter dysfunction and unplanned removal but may also trigger serious complications such as pulmonary embolism and post-thrombotic syndrome, thereby prolonging hospital stays, increasing medical costs, and even threatening patients' lives (2). Cancer patients inherently exhibit a hypercoagulable state due to factors such as the release of procoagulant substances by tumor cells and the body's chronic inflammatory state. The placement of CVADs may further impair vascular endothelial integrity and disrupt local haemodynamics, thereby significantly increasing the risk of CRT (3).

In recent years, researchers in China and internationally have conducted numerous studies on the risk factors for CRT in cancer patients, suggesting that patient age, underlying conditions, tumor characteristics, catheter type, and treatment regimens may be associated with CRT. However, existing studies show inconsistencies in the identification of these factors and lack a systematic synthesis and analysis, making it difficult to comprehensively characterize the spectrum of risk factors for CRT in cancer patients (4). Therefore, this study used a systematic review methodology to comprehensively collect relevant studies from China and other countries, synthesize and analyze the risk factors for CRT in cancer patients, and provide evidence-based support for the development of scientifically effective prevention strategies and the reduction of CRT incidence in clinical practice.

## 2 Methods

### 2.1 Search strategy

This study conducted a systematic search of PubMed, Embase, Web of Science, and the Cochrane Library from database inception to February 2026. The search focused on studies related to catheter-related thrombosis in cancer patients. A combination of subject headings and free-text terms was used. The search terms included “neoplasms”, “cancer”, “oncology”, “tumor”, “tumour”,“implantable venous port”, “VAP”, “IVAP”, “VPA”, “TIVAP”, “CVPAS”, “port”, “peripherally inserted central catheter”, “PICC”, “vein thrombosis”, “venous thromboembolism”, “VTE”, “VT”, “risk factor”, “factor”, and “risk”, which were combined using the Boolean operators AND and OR. In addition, relevant studies were identified by manually reviewing the reference lists of included articles and relevant reviews. Two authors independently screened the titles, abstracts, and full texts of the retrieved articles, and any discrepancies were resolved through discussion or consultation with a third reviewer.

### 2.2 Study selection

#### 2.2.1 Inclusion criteria

1. Study designs: prospective and retrospective cohort studies, case-control studies, cross-sectional studies, and randomized controlled trials.
2. Study population: cancer patients who had undergone CVAD insertion, including PICCs, implantable infusion ports, and tunnelled central venous catheters.
3. Outcomes: studies reporting the occurrence of CRT and clearly specifying the diagnostic methods used for its confirmation.

#### 2.2.2 Exclusion criteria

1. Study designs: case reports, reviews, expert opinions, conference abstracts, animal studies, or *in vitro* experiments.
2. Study population/outcomes: studies involving non-cancer patients, studies not involving CVADs, or studies that did not report CRT or analyze its risk factors.
3. Incomplete data: studies with incomplete data or studies for which the full text or essential data could not be obtained.
4. Duplicate publications.

### 2.3 Data extraction

To ensure the accuracy and reliability of the data collected for this review, data extraction was performed independently by two authors, who cross-checked all extracted information. Discrepancies in the extracted data were resolved through discussion and re-evaluation. Where consensus could not be reached, a third researcher was consulted until agreement was achieved. The extracted information included the first author and year of publication, study population, sample size, diagnostic methods for CRT, risk factors, odds ratios (ORs) or hazard ratios (HRs) with 95% confidence intervals (CIs), coefficients, and *p*-values. Where data were missing, the study authors were contacted.

### 2.4 Data synthesis

Meta-analysis was not undertaken because the included studies showed substantial clinical and methodological heterogeneity. They varied in cancer type and stage, patient age, catheter type and insertion setting, CRT diagnostic methods, follow-up duration, and the definitions and thresholds applied to risk factors. The reported estimates also differed in effect measures, reference categories, and covariates included in multivariable models. Taken together, these differences did not support a reliable or clinically meaningful pooled estimate. A structured narrative synthesis was therefore performed. Study characteristics and reported associations were first tabulated, after which risk factors were organised into patient-related, catheter-related, and treatment-related domains. When the same or a closely related factor was examined in more than one study, the direction and consistency of its association with CRT were compared across studies.

## 3 Results

### 3.1 Literature search

An initial database search retrieved 15,372 records. After removing 654 duplicates and excluding 14,544 records flagged ineligible by automated tools, 174 records proceeded to title-abstract screening. Fifty-two records were excluded at this stage. Full-text assessment was pursued for 122 records, of which 22 were unobtainable. Of the remaining 100 full-text articles, 42 were excluded for ineligibility. Ultimately, 58 studies enrolling 50,987 cancer patients were included. The literature screening process is illustrated in Figure 1.

**Figure 1:**
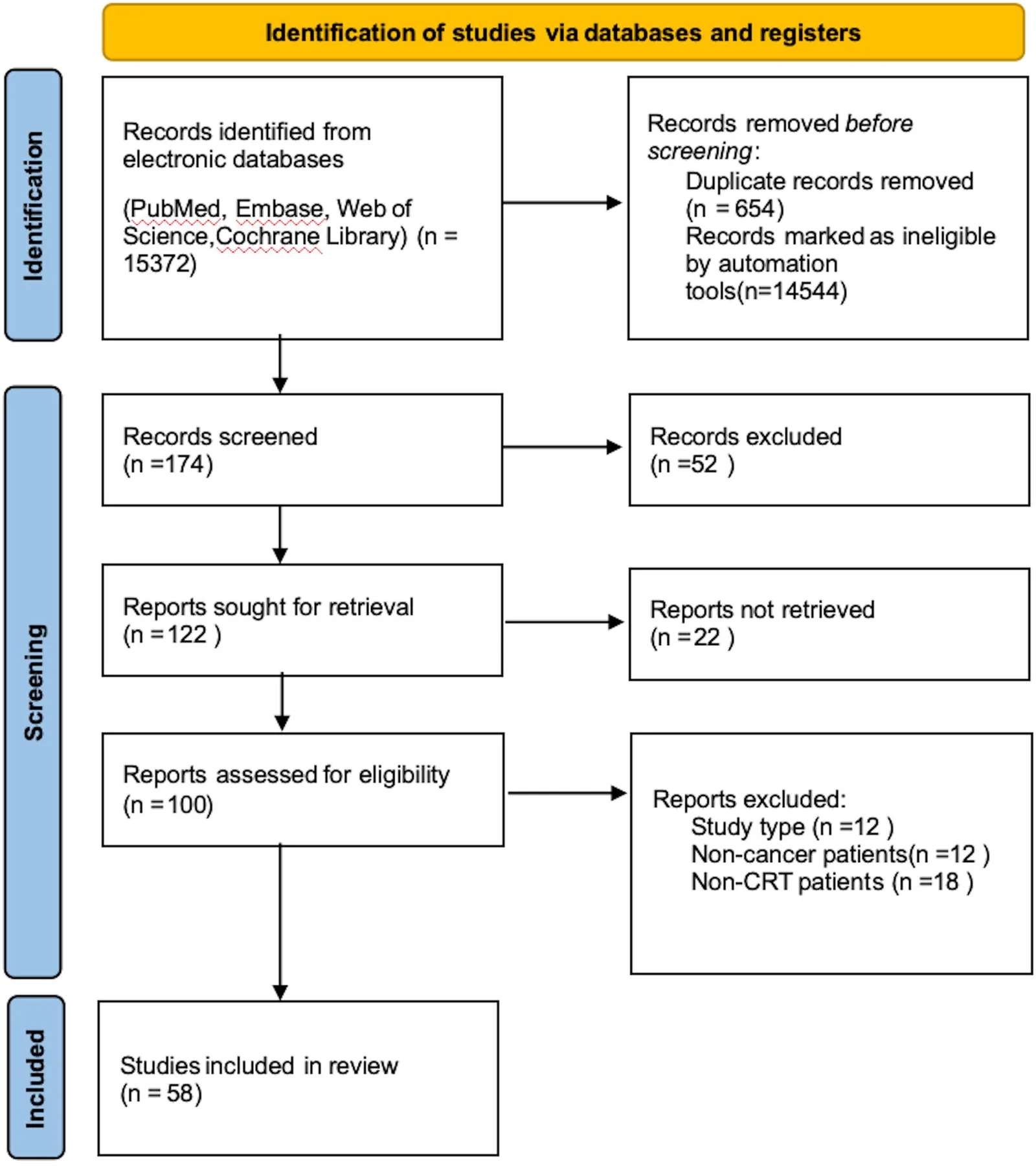
Screening flowchart.

### 3.2 Characteristics of the included studies

The patient-related, catheter-related, and treatment-related factors associated with CRT are summarised in Tables 1–3. Detailed characteristics of the included studies are provided in Supplementary Table S1. These studies were published between 1993 and 2025. Various imaging modalities were used to diagnose CRT, including ultrasonography and colour Doppler ultrasound.

**Table 1:** Summary of patient-related factors associated with CRT.

Subcategory	Specific factor	Reference(s)
Demographic characteristics	Older age^b^	(5–8, 17, 18, 46, 53, 55)
	Male sex	(5, 8–11, 17, 52)
Underlying conditions and medical history	Previous thrombosis	(13, 17–19, 24, 25, 37, 61)
	Positive family history of thrombosis	(20)
	Diabetes	(7, 10, 12, 18, 21, 22, 60)
	Hypertension^b^	(8, 10)
	Chronic obstructive pulmonary disease	(23)
	Upper-limb lymphoedema	(23, 32)
	Comorbidities	(11, 26, 27)
	Hepatitis B virus infection	(28)
	Sepsis^a^	(9)
	Hyperlipidaemia	(61)
Tumor-related characteristics	Tumor type	(5, 6, 15, 29, 31)
	Advanced tumor stage^b^	(7, 18, 26, 61)
	Metastatic disease	(7, 14, 21, 24, 48, 52)
	Larger tumor size	(28)
	Adenocarcinoma or lobular histology	(27, 30)
	High or very high cancer thrombosis risk category	(34)
Physiological and laboratory indicators	Menopause	(23)
	Anaemia	(12)
	Myelosuppression	(7)
	Elevated platelet count	(10, 23, 32, 42)
	Elevated D-dimer	(12, 18, 23, 25, 32–35, 61)
	Shortened activated partial thromboplastin time	(5, 23)
	Shortened prothrombin time^b^	(10, 36, 37)
	Low antithrombin III level	(25, 38)
	Elevated fibrinogen	(47)
	Shortened thromboelastography R and K values	(10)
	Factor V G1691A heterozygosity	(39)
	Elevated white blood cell count	(5)
	Higher haemoglobin level	(10)
	Elevated total cholesterol	(25)
	Elevated triglycerides	(29, 41)
	Elevated low-density lipoprotein cholesterol	(28)
	Non-O blood group (A, B, or AB)	(17, 41)
	Reduced blood flow velocity	(34, 40)
	Larger arm circumference	(29)
	Higher Caprini thrombosis risk score	(37, 47)
Lifestyle and physical condition	Reduced physical activity	(10, 11, 22, 43, 60)
	Higher body mass index or obesity^b^	(27, 34, 36, 43–45)
	Smoking	(33, 45)
	Poor performance status or impaired self-care	(7, 11, 24, 35, 40)
	Shorter height	(35)
	Severe coughing	(46)
	Severe vomiting	(46)

Unmarked factors were associated with a higher risk of CRT.

CRT, catheter-related thrombosis.

a Factors were associated with a lower risk of CRT.

b Both higher- and lower-risk associations were reported across studies.

**Table 2:** Summary of catheter-related factors associated with CRT.

Subcategory	Specific factor	Reference(s)
Catheter placement characteristics	Tertiary or specialised hospital setting	(6)
	Previous central or deep venous catheterisation	(6, 11, 23, 32, 44, 46)
	Multiple insertion attempts	(11, 18, 31, 35, 37)
	Insertion method^a^	(12)
	Left-sided, subclavian, or cephalic-vein insertion^b^	(48–51, 54)
	Improper catheter-tip position	(15, 18, 25, 48, 52)
	Shallow vessel depth^a^	(53)
	Smaller or specific vessel diameter^b^	(29, 53)
	Catheter-to-vessel diameter ratio >0.35	(41)
	Greater catheter position-related distance	(42)
	Aspiration difficulty or ball-valve effect	(49)
Catheter characteristics	PICC use (vs other vascular access devices)	(9, 13, 15, 44, 56, 57)
	Catheter material^b^	(5, 12)
	Open-ended catheter	(5, 6)
	Larger catheter size	(33, 58)
	Catheter length^a^	(12)
	Ceramic port reservoir	(26)
Factors related to catheter placement and use	Catheter dwell time^b^	(5, 7)
	Reduced activity of the catheterised limb	(11, 60)
	Catheter occlusion or treatment for occlusion^b^	(9, 31)
	Catheter-related infection	(12, 19, 59)
	Non-infectious catheter complications	(19)
	Repeated catheterisation	(56)
	Greater thrombus extent	(38)

Unmarked factors were associated with a higher risk of CRT.

CRT, catheter-related thrombosis.

a Factors were associated with a lower risk of CRT.

b Both higher- and lower-risk associations were reported across studies.

**Table 3:** Summary of treatment-related factors associated with CRT.

Subcategory	Specific factor	Reference(s)
Antineoplastic treatment modalities	Surgery^b^	(10, 12, 23, 30, 32, 60, 61)
	Chemotherapy^b^	(6, 8, 15, 22, 26, 37)
	Targeted therapy	(12)
	Thoracic radiotherapy	(48)
Medication use	Cisplatin	(5)
	Vincristine	(5)
	Carboplatin	(33)
	Docetaxel	(33)
	Paclitaxel plus cisplatin	(37)
	Gemcitabine plus cisplatin	(47)
	Fluorouracil-containing chemotherapy	(8)
	Hormonal therapy	(61)
	Endocrine therapy	(23)
	Anticoagulant therapy^b^	(18, 59, 62)
	Antiplatelet therapy^a^	(15, 54, 59)
	Heparin catheter care^a^	(48)
	Erythropoiesis-stimulating agents	(59)
Treatment-related procedures and adjunctive therapies	Parenteral nutrition	(7)
	Catheter removal before anticoagulation	(16)
	Catheter reinsertion after removal	(16)
	Hospitalisation	(59)
	Concurrent infection	(5, 12, 61)

Unmarked factors were associated with a higher risk of CRT.

CRT, catheter-related thrombosis.

a Factors were associated with a lower risk of CRT.

b Both higher- and lower-risk associations were reported across studies.

### 3.3 Risk factors for CRT in cancer patients

Risk factors for CRT in patients with cancer were grouped into three domains:

patient-related, catheter-related, and treatment-related factors, as shown in Figure 2.

**Figure 2:**
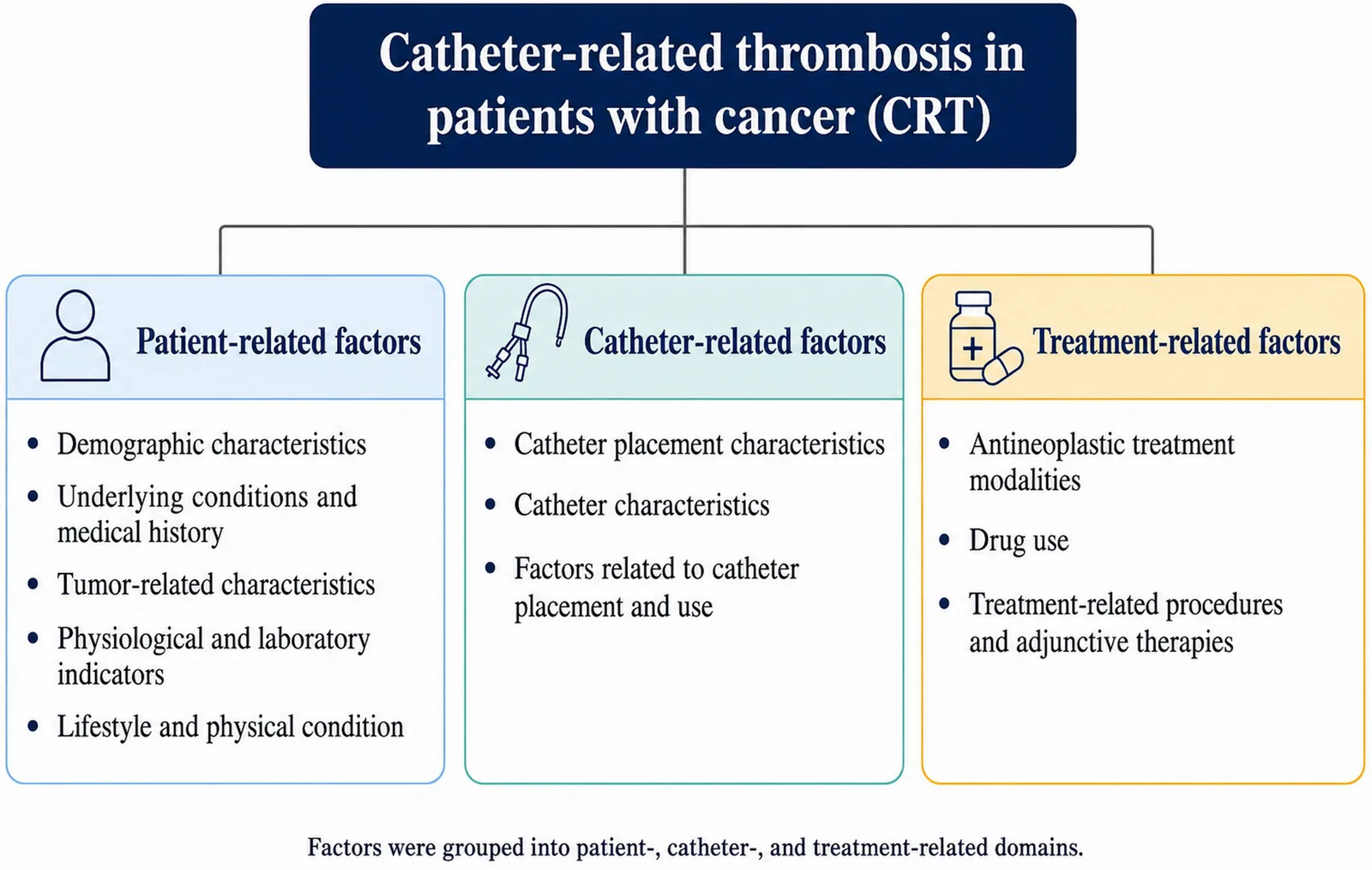
Summary of factors associated with catheter-related thrombosis in patients with cancer.

#### 3.3.1 Patient-related factors

The 58 studies showed that patient-related factors were among the major contributors to CRT and could be categorised into five sub-groups: demographic characteristics, underlying diseases and medical history, tumor-related characteristics, physiological and laboratory indicators, and lifestyle and physical condition.

##### 3.3.1.1 Demographic characteristics

Demographic correlates of CRT were synthesised from 12 studies. Nine studies assessed age-related effects using diverse analytical strategies. Categorical cut-offs including age >65 years (5, 6), age ≥60 years (7, 8), and older age at cancer diagnosis predicted higher CRT risk (9). Continuous-variable analyses reported elevated risk per one-year age increment (10–12). Nevertheless, M. L. Ellis, S 2020 reported countervailing findings: both 50-61-year and >61-year subgroups exhibited lower CRT risk relative to patients aged <50 years, reflecting notable population-level heterogeneity (13).

Male sex increased CRT risk among PICC and port recipients (5, 6, 10, 14, 15). In patients with symptomatic upper-extremity deep vein thrombosis, male sex also predicted 3-month thrombotic recurrence (16).

##### 3.3.1.2 Underlying conditions and medical history

Twenty-three studies reported that underlying conditions and medical history were closely associated with CRT. Previous thrombotic events, cardiometabolic disorders, and selected chronic conditions formed the main groups of relevant factors. A history of venous thromboembolism or thrombosis was repeatedly identified in general oncology, chemotherapy, and haematopoietic stem cell transplantation settings (17–19); a positive family history of thrombosis was also relevant in pediatric and adolescent cancer populations (20). Diabetes was reported across several cancer and catheter cohorts as a risk factor for CRT (10, 12, 18, 21, 22). For hypertension, inconsistent findings were observed: hypertension was an independent risk factor for CRT (10), whereas drug-controlled hypertension was identified as inversely associated with CRT (8). Chronic obstructive pulmonary disease and upper-limb lymphoedema were further independent risk factors among patients with breast cancer (23).

Notably, an extreme effect size was observed in nasopharyngeal carcinoma cohorts: a prior history of venous thromboembolism was associated with a markedly elevated CRT risk (24), and a history of thrombosis within the past 12 months also represented a strong risk factor in lymphoma populations (25). In terms of comorbidity burden, an increased number of comorbidities was associated with higher odds of asymptomatic CRT in breast cancer patients (26), and the presence of at least one comorbidity was linked to symptomatic CRT in non-metastatic breast cancer cohorts (27). Hepatitis B virus infection (HBsAg positivity) was also identified as a risk factor in early-stage breast cancer populations (28).

##### 3.3.1.3 Tumor-related characteristics

Eighteen studies reported that tumor type, stage, and metastatic status were associated with CRT. Associations were reported for gastrointestinal, lung or thoracic, breast, head and neck, genital tract, and haematological malignancies, although the reference cancer category and estimated effect size differed across cohorts (5, 6, 15, 29). Advanced disease, especially stage III–IV cancer and distant or M-stage metastasis, showed a more consistent association across diverse cancer populations and catheter types (7, 12, 14, 18, 21).

In site-specific analyses, metastatic M-stage disease was confirmed as an independent risk factor in nasopharyngeal carcinoma patients (24), and tumor size exceeding 2 cm was associated with elevated CRT risk in early-stage breast cancer (28). In breast cancer cohorts, menopause represented a tumor-associated endocrine risk factor for CRT (23). Histological subtypes also influenced risk: lobular carcinoma histology increased CRT risk in non-metastatic invasive breast cancer (27), and adenocarcinoma histology was linked to higher risk in esophageal cancer cohorts (30). Ovarian cancer was associated with a particularly elevated risk relative to other tumor types (31).

##### 3.3.1.4 Physiological and laboratory indicators

Twenty-three studies reported that coagulation, haematological, and biochemical indicators were associated with CRT. Platelet-related measures and elevated D-dimer were the most frequently reported markers of increased CRT risk, with a consistent dose-response relationship observed across multiple cohorts (10, 12, 18, 23, 25, 32–35). Heterogeneous associations were observed among coagulation parameters. Shortened activated partial thromboplastin time and shortened prothrombin time were identified as risk factors for CRT, whereas prolonged prothrombin time (PT), as well as prolonged thromboelastography-R and K values, were associated with lower CRT risk, suggesting that a relatively hypocoagulable state correlates with lower CRT risk (5, 10, 23, 36, 37). Higher antithrombin III levels were associated with lower CRT risk, while antithrombin III deficiency was linked to increased thrombus severity (25, 38). Heterozygous carriage of the factor V G1691A mutation was associated with elevated CRT risk in female breast cancer patients (39).

A white blood cell count above normal, haemoglobin level, and reduced peripheral blood flow velocity were also associated with CRT occurrence (5, 10, 34, 40). Additionally, anaemia and myelosuppression, as laboratory-confirmed haematological abnormalities, were associated with elevated CRT risk (7, 12). Metabolic and anthropometric indicators included elevated triglycerides, total cholesterol and LDL cholesterol, arm circumference, and non-O ABO blood groups (17, 25, 28, 29, 41). Elevated platelet count was also verified as a risk factor in pediatric oncology populations (42). Higher cancer thrombosis risk stratification scores correlated with progressively elevated CRT risk (34).

##### 3.3.1.5 Lifestyle and physical condition

Sixteen studies reported that physical activity, physical fitness, and lifestyle habits were associated with CRT. The evidence on activity level was not uniform: reduced activity or physical inactivity was identified as a risk factor in multiple cohorts (10, 22, 43). Frequent physical activity was associated with significantly lower CRT risk in lymphoma cohorts (25). Poor functional status, reflected by a Karnofsky Performance Status score ≤50, impaired self-care ability, physical activity disorder, and worse ECOG performance scores, was consistently associated with elevated CRT risk (7, 10, 24, 35, 40).

Elevated BMI and obesity were associated with higher CRT risk across most adult solid tumor and haematological malignancy cohorts (27, 34, 36, 44, 45), whereas an inverse association was reported in nasopharyngeal carcinoma patients (24). Smoking was confirmed as a lifestyle-related risk factor, and acute physiological stressors such as vigorous coughing and severe vomiting also increased CRT risk (45, 46). Higher Caprini thrombosis risk scores were associated with elevated CRT odds (37, 47).

#### 3.3.2 Catheter-related factors

Thirty-six studies reported that catheter-related factors were important contributors to CRT and could be categorised into three subgroups: catheter placement characteristics, catheter properties, and factors related to catheter placement and use.

##### 3.3.2.1 Catheter placement characteristics

Twenty-three studies reported that placement site, placement technique, number of insertion attempts, and vascular conditions were associated with CRT. Previous central or deep venous catheterisation and repeated insertion attempts were recurring procedural risk factors (6, 11, 12, 18, 23, 31, 32, 35, 37). Heterogeneous findings existed regarding catheter insertion side; left-sided access was reported as a risk factor for CRT in several studies, while one study identified left-sided catheter placement as inversely associated with CRT (26, 48–51). Improper tip positioning represented an important risk factor for CRT, whereas appropriate catheter tip position was inversely associated with CRT (15, 18, 25, 31, 48, 49, 52). Aspiration difficulty or a ball-valve effect, and catheter blockage characterised technically problematic placements associated with thrombosis (15, 18, 31, 48, 49, 52). For vascular-related indicators, the PICC-to-vessel diameter ratio >0.35 served as a risk factor for CRT. Some vascular parameters, including particular catheterisation method, selected catheter specifications, smaller post-insertion vessel diameter and shallower vessel depth <1.0 cm, showed inverse correlations with CRT; nevertheless, inconsistent results were observed for certain vessel diameter ranges (12, 17, 29, 41, 42, 53). The vertical distance between the catheter's highest point and sternoclavicular landmarks was also correlated with CRT occurrence (41). Hospital level and hospital nature were also identified as contextual factors related to placement quality and CRT risk (6).

Regarding insertion vein selection, use of the cephalic vein for port catheter placement was associated with a significantly higher CRT risk compared with jugular or subclavian vein access (54), highlighting the importance of venous access site selection beyond left right laterality.

##### 3.3.2.2 Catheter characteristics

Twelve studies reported that catheter-specific characteristics, such as type, material, and size, were associated with CRT. Catheter or PICC type was repeatedly associated with CRT across pediatric, haematological, and general oncology populations (9, 13, 15, 44, 55–57). Relevant design and material features linked to higher CRT risk included polyurethane and open-ended PICCs, as well as ceramic-base port reservoirs (5, 6, 26). Nevertheless, certain catheter materials were associated with reduced odds of CRT (12). Larger 5-Fr and 6-Fr PICC sizes were also associated with increased CRT risk (33, 58).

##### 3.3.2.3 Factors related to catheter placement and use

Eleven studies reported that catheter dwell time, occlusion-related management, and infection were associated with CRT. Longer dwell time was generally associated with elevated CRT risk, with risk increasing alongside prolonged indwelling duration (5). However, subgroup analysis from one included study indicated that relatively prolonged indwelling periods (1-6 months and >6 months) were inversely associated with CRT (7). Catheter-related infection was consistently linked to higher CRT risk across different clinical settings (12, 19, 59). Reduced activity of the catheterised limb was another notable modifiable risk factor (11, 60). For occlusion-related variables, while repeated catheterisation procedures and greater thrombus extent increased CRT risk, administration of tPA for CVC occlusion was associated with lower odds of CRT (9, 38, 56). Non-infectious catheter complications also showed an association with CRT occurrence (19).

#### 3.3.3 Treatment-related factors

Twenty-three studies reported that treatment-related factors were closely associated with CRT and could be categorised into three subgroups: antineoplastic treatment modalities, drug use, and treatment-related procedures and adjuvant therapy.

##### 3.3.3.1 Antineoplastic treatment modalities

Fourteen studies reported that surgery, chemotherapy, radiotherapy, and other treatment modalities were associated with CRT. Surgical factors associated with higher risk included a history of surgery, recent surgery within 1 month, major procedures lasting >45 min, and neck lymph node dissection (10, 12, 30, 60, 61). Nevertheless, among breast-cancer populations, breast surgery was inversely associated with CRT (23, 32). Chemotherapy-related findings were heterogeneous: whereas prior or current chemotherapy, exposure to systemic chemotherapy drugs, and the initiation of chemotherapy or targeted therapy at enrolment generally increased CRT risk (6, 15, 22, 37), subgroup analyses focusing on symptomatic CRT demonstrated that chemotherapy was inversely associated with symptomatic CRT (26). Thoracic radiotherapy was also associated with significantly increased CRT risk (48). In breast-cancer cohorts, endocrine therapy was linked to elevated thrombosis risk (23, 61).

##### 3.3.3.2 Drug use

Thirteen studies reported that chemotherapy drugs, anticoagulants, hormones, and other agents were associated with CRT. Medication-related associations involved specific cytotoxic regimens, hormonal or targeted treatments, antithrombotic therapy, and erythropoiesis-stimulating agents. Reported cytotoxic agents or combinations linked to higher CRT risk included cisplatin, vincristine, carboplatin, docetaxel, paclitaxel plus cisplatin, gemcitabine plus cisplatin, and fluorouracil-containing regimens (5, 33, 47). Hormone therapy and targeted therapy were also associated with increased CRT risk (12, 61). Baseline anticoagulant and antiplatelet exposure were associated with lower CRT risk across multiple cohorts (15, 18, 54, 59), whereas treatment-dose anticoagulation showed a positive association with CRT, likely reflecting confounding by indication. Erythropoiesis-stimulating agents were identified as a strong medication-related risk factor for CRT (59).

Notably, confounding by indication was further supported by real-world data from solid tumor chemotherapy cohorts, where anticoagulant therapy was associated with elevated CRT risk (62), consistent with the pattern observed for treatment-dose anticoagulation.

##### 3.3.3.3 Treatment-related procedures and adjunctive therapies

Six studies reported that treatment-related procedures and adjunctive therapies were associated with CRT. Parenteral nutrition and hospitalisation were associated with significantly elevated CRT risk (7, 59). Catheter removal before anticoagulation followed by reinsertion was linked to increased venous thromboembolism recurrence within 3 months and at 1 year of follow-up (16). Concurrent systemic or local infection was an additional recurring factor that amplified CRT risk (5, 61).

## 4 Discussion

This systematic review integrated data from 58 high-quality clinical studies involving 50,987 cancer patients and systematically identified three major categories of risk factors for catheter-related thrombosis (CRT): patient-related, catheter-related, and treatment-related factors. Each category comprised multidimensional sub-indicators, and many of these factors were identified as statistically significant in multivariate analyses. Notably, several factors demonstrated bidirectional associations depending on clinical context, with some variables showing inverse associations with CRT in specific populations. Collectively, these findings provide an evidence base for stratified CRT prevention and comprehensive catheter management in clinical practice. The following discussion integrates the direction, magnitude, and clinical relevance of the reported associations while considering the strengths and limitations of the available evidence, with the aim of informing clinical practice and future research.

### 4.1 Patient-related factors

Patient-related factors, as major intrinsic contributors to CRT, encompass five dimensions: demographic characteristics, underlying conditions and medical history, tumor-related characteristics, physiological and laboratory indicators, and lifestyle and physical condition. These factors may interact and collectively influence a patient's risk of thrombosis. Among the demographic factors, older age—most commonly defined using a cutoff of 60 or 65 years—and male sex were nonmodifiable factors associated with higher CRT risk, with reported OR of 1.791 for age over 65 years (5) and 2.331 for male sex (6). These findings support prioritizing these groups for early CRT risk assessment and closer monitoring.

Among underlying conditions and medical history, a history of venous thromboembolism, diabetes, and hypertension were important clinical risk factors for CRT. Across multivariable analyses, the reported ORs were 1.987 for a history of thrombosis (17), 2.78 for a previous thromboembolic event before autologous stem cell transplantation (19), 2.478 for diabetes, and 2.386 for hypertension (10), indicating consistent associations between thrombotic history, cardiometabolic comorbidities, and CRT risk. These conditions may increase the risk of thrombosis through coagulation abnormalities and endothelial dysfunction. However, inconsistent findings were observed for hypertension: whereas uncontrolled hypertension was a risk factor, drug-controlled hypertension was identified as inversely associated with CRT (8), underscoring the importance of active blood pressure management. Clinically, active control of blood glucose and blood pressure, together with strengthened anticoagulation monitoring in patients with a history of thrombosis, may help reduce the incidence of CRT.

Tumor-related characteristics were also strongly associated with CRT risk. Specific tumor types, including lung cancer, gastrointestinal tumors, and head and neck tumors, as well as advanced disease features such as stage III–IV disease and distant metastasis, were associated with a significantly increased risk of thrombosis. This may be related to the chronic inflammatory state of advanced cancer and the release of procoagulant substances by tumor cells, which further aggravate hypercoagulability.

Physiological and laboratory parameters are important objective indicators for CRT risk assessment. Elevated platelet counts, D-dimer >0.55 mg/L, shortened activated partial thromboplastin time, and other coagulation abnormalities, together with triglycerides ≥1.7 mmol/L and blood groups A, AB, or B, have been reported as risk factors for CRT. Representative multivariable estimates included an OR of 3.428 for D-dimer over 0.55 mg/L and an OR of 2.072 for blood type A (17, 32). Conversely, several coagulation parameters showed inverse associations with CRT risk:prolonged prothrombin time, higher thromboelastography R and K values (10), and higher antithrombin III levels were associated with reduced CRT risk (25), reflecting that adequate endogenous anticoagulant capacity correlates with lower thrombotic risk. These findings suggest that routine laboratory monitoring may facilitate early identification of thrombotic warning signs. Across the lifestyle and functional domains, reduced physical activity, smoking, obesity, and poor performance status were associated with higher CRT risk. Reported ORs were 3.90 for smoking, 1.11 for BMI at least 30 kg/m², and 2.045 for a Karnofsky Performance Status score no higher than 50 (7, 45). In contrast, frequent physical activity was associated with substantially lower CRT risk (61), indicating that encouraging physical activity, smoking cessation, and functional improvement may help reduce the risk of thrombosis.

### 4.2 Catheter-related factors

Catheter-related factors are important modifiable targets for CRT prevention. These can be categorised into three sub-groups: catheter placement characteristics, catheter properties, and factors related to catheter placement and use. Their associations with CRT may reflect the standardization of placement procedures, compatibility between catheter characteristics and vascular anatomy, and the quality of catheter management. In clinical practice, these factors may be addressed through optimised catheter selection, standardised placement procedures, and strengthened maintenance across the entire catheterization process.

Across studies, left upper-limb placement, two or more insertion attempts, and inappropriate catheter tip positioning were associated with higher CRT risk, with reported ORs of 2.29, 9.457, and 4.05, respectively (11, 48). However, heterogeneous findings existed regarding insertion side: left-sided catheter placement was inversely associated with CRT in a breast cancer cohort (26), suggesting that the effect of insertion side may vary by tumor type and vascular anatomy. Similarly, whereas improper tip positioning was a consistent risk factor, appropriate catheter tip position was strongly associated with reduced CRT risk (25). These findings may be explained by slower venous flow in the left upper limb, repeated endothelial injury from multiple punctures, and hemodynamic disturbance caused by malpositioned catheter tips. Clinically, priority should be given to optimal insertion sites such as the right upper limb or internal jugular vein when clinically appropriate, with ultrasound guidance used whenever possible to minimize puncture attempts and ensure accurate tip placement at the cavoatrial junction. In addition, matching catheter size to vessel caliber and avoiding a PICC-to-vessel ratio over 0.35 may help reduce venous stasis and CRT risk.

Comparative evidence from one multivariate Cox regression study demonstrated that PICC use was associated with substantially higher CRT risk compared with implantable ports, with a reported HR of 22.2 [2.9-170.6] (13). Further evidence identified polyurethane material, a catheter diameter of at least 5 Fr, and an open-ended design as higher-risk characteristics relative to silicone, smaller-diameter, and valved alternatives. Reported multivariable ORs were 3.524 for a 5-Fr PICC, and 2.449 for a polyurethane PICC (5, 33). Conversely, silicone catheter material and valved catheter design showed inverse associations with CRT risk (12). These findings suggest that implantable ports may be preferred when clinically appropriate for patients requiring long-term vascular access. If a PICC is used, a silicone-based, small-diameter, valved catheter may be more appropriate. Catheter selection should be individualized based on vascular condition and treatment needs to optimize compatibility with the patient's vasculature.

Across the available evidence, prolonged catheter dwell time, catheter occlusion, and infection were consistently associated with higher CRT risk. Reported estimates included an OR of 1.003 for each additional day of catheter dwell time (5). Catheter-related infection was consistently linked to higher CRT risk across settings, with ORs of 7.619 and 2.88 reported in general oncology and multiple myeloma cohorts, respectively (5, 19). However, a subgroup analysis in a liver cancer cohort found that relatively prolonged indwelling periods were inversely associated with CRT (7), possibly reflecting survivor bias or differences in catheter management. These findings may be explained by chronic endothelial stimulation from long-term indwelling, venous stasis due to occlusion, and infection-related activation of the coagulation system. Clinically, routine catheter maintenance should be strengthened, including timely flushing, prevention of occlusion, regular monitoring for catheter-related infections, and reassessment of the ongoing need for the catheter. Catheters that are no longer needed should be removed promptly, and patients should be advised to maintain appropriate movement of the catheterized limb to preserve local blood flow.

### 4.3 Treatment-related factors

Associations between treatment-related factors and CRT may reflect the effects of anticancer therapy on coagulation, vascular endothelial function, hemodynamics, and the coagulation–fibrinolytic balance. These factors can be categorised into three major subcategories: anticancer treatment modalities, drug use, and treatment-related procedures and adjuvant therapies. Clinically, these factors can be appropriately addressed through optimised treatment regimens, standardised treatment procedures, and rational drug use, thereby reducing the risk of CRT whilst ensuring the efficacy of anticancer treatment.

Across studies, recent surgery, chemotherapy, and thoracic radiotherapy were associated with increased CRT risk. Reported ORs were 2.258 for surgery within the previous month, 8.982 for a history of operation, and 7.01 for thoracic radiotherapy (10, 12, 48). However, among breast cancer populations, breast surgery was inversely associated with CRT (23, 32), likely reflecting improved postoperative mobilization and reduced local tumor burden. Chemotherapy-related findings were similarly heterogeneous: whereas systemic chemotherapy generally increased CRT risk (6), subgroup analysis focusing on symptomatic CRT demonstrated that chemotherapy was inversely associated with symptomatic CRT (26). These associations may be explained by surgery-related stress responses, chemotherapy-induced endothelial stimulation, and radiation-related vascular inflammation, all of which may aggravate endothelial injury and hypercoagulability. Clinically, early postoperative mobilization and physical prophylactic measures, such as pneumatic compression, should be considered for surgical patients. For patients receiving chemotherapy or thoracic radiotherapy, vascular protection, thrombosis monitoring, and dynamic CRT risk assessment are warranted throughout the treatment cycle.

Across chemotherapy studies, regimens containing fluorouracil, cisplatin, vincristine, carboplatin, or docetaxel were associated with increased CRT risk. Representative ORs were 2.347 for cisplatin, 2.329 for vincristine, 2.429 for fluorouracil-containing regimens, and 7.227 for docetaxel (5, 8, 33). Hormone therapy and targeted therapy were also associated with increased CRT risk, with reported ORs of 2.47 and 1.631, respectively (12, 61). These findings suggest that, when formulating chemotherapy regimens, clinicians should give greater attention to CRT risk in patients receiving agents with higher thrombotic potential while balancing antitumor efficacy. When hormones or targeted therapies are used, drug dosage and treatment duration should be determined carefully in conjunction with the patient's thrombosis risk stratification, and thromboprophylaxis may be considered when clinically indicated. Baseline anticoagulant or antiplatelet use showed an inverse association with CRT (15), and anticoagulant therapy was similarly associated with lower CRT risk (18); however, these observational findings do not establish a routine prophylactic benefit and must be weighed against the risk of bleeding. Treatment should therefore be individualized according to the patient's clinical circumstances, and inappropriate medication use should be avoided. In addition, erythropoiesis-stimulating agents may further increase CRT risk, with a reported OR of 10.66 (59), supporting cautious use based on strict clinical indications.

### 4.4 Strengths and limitations

As a systematic review examining the risk factors for CRT in cancer patients, this study has several notable methodological strengths. First, the search scope was comprehensive, covering four major databases—PubMed, Embase, Web of Science, and the Cochrane Library—and extending to February 2026. The study included 58 studies and 50,987 participants, representing a large sample size. It encompassed clinical data from different regions, tumor types, and study designs, which enhances the generalisability and reliability of the findings. Second, the methodology was rigorous and followed the methodological requirements for systematic reviews. Three researchers assessed the inclusion and exclusion criteria, whilst two researchers independently screened the literature and extracted data, resolving discrepancies through consensus, thereby effectively minimizing selection bias and information bias. Third, the risk factors were clearly categorised, with the determinants of CRT systematically divided into three major categories and over ten subcategories. Each factor was accompanied by explicit supporting evidence and statistical indicators, including ORs/HRs, 95% CIs, and p-values, providing clear evidence for clinicians to identify high-risk factors and formulate stratified prevention strategies accurately.

However, this study also has certain limitations that require attention in clinical application and future research. First, some of the included studies were retrospective, which inevitably introduced recall bias and selection bias and may have affected the estimation of effect sizes for certain factors. Second, there were variations in the diagnostic criteria for CRT and in the duration of follow-up across studies. Some studies used ultrasound for diagnosis, whilst others combined CT angiography and intravenous angiography. The follow-up duration also varied, which may have affected the consistency of the results. Third, variation in study populations, catheter types, CRT diagnostic methods, follow-up periods, risk-factor definitions, and analytical approaches limited direct comparison across studies. Pooled estimates could therefore not be derived reliably, and the strength of associations within specific patient or catheter subgroups remains uncertain. Although the narrative synthesis offered a structured overview of the available evidence, it did not produce an overall effect estimate or quantify between-study heterogeneity. Future studies using consistent definitions, outcome measures, and reporting standards may make quantitative synthesis possible. Fourth, the included studies lacked specific investigations of CRT risk factors in particular cancer subtypes, such as pancreatic and ovarian cancers, and relevant data were limited for special cancer populations, such as children and adolescents, making it difficult to provide more targeted evidence-based guidance. Fifth, the mechanisms underlying the interactions between certain influencing factors remain unclear. For instance, the synergistic effects of underlying conditions and tumor characteristics, or of chemotherapy drugs and catheter types, on CRT were not explored in depth in this study, thereby limiting the provision of a stronger theoretical basis for the development of combined intervention strategies in clinical practice.

Notably, substantial clinical heterogeneity existed across multiple risk-related factors in the included studies. For instance, whilst general surgical history represented a risk factor for CRT (61), breast-cancer-related surgery was inversely associated with CRT in another cohort (23). Similar conflicting associations were also observed for several other variables, including hypertension, left-sided catheter insertion and catheter dwell time. Such discrepancies may arise from variations in study populations, tumour subtypes, catheter characteristics and CRT diagnostic criteria. Given this considerable clinical heterogeneity across numerous influencing factors, we undertook a qualitative synthesis rather than pooling effect-sizes via quantitative meta-analysis.

## 5 Conclusion

The risk factors for CRT in cancer patients are complex and diverse and can be broadly classified into patient-related, catheter-related, and treatment-related factors. Clinicians should stratify patients according to these risk factors and implement individualised preventive measures, including appropriate catheter selection and insertion-site choice, standardised catheterization and maintenance procedures, active management of underlying conditions and infections, and optimisation of treatment regimens, to reduce the incidence of CRT and improve patient outcomes. In the future, more high-quality prospective cohort studies are needed to further validate the magnitude of effects of different risk factors and to inform the development of more precise CRT prevention strategies.

## References

[1] Laguna JC Cooksley T Ahn S Tsoukalas N Oo TH Brito-Dellan N. Catheter-related thrombosis (CRT) in patients with solid tumors: a narrative review and clinical guidance for daily care. Support Care Cancer. (2022) 30(10):8577–88. 10.1007/s00520-022-07297-8 35932317

[2] Marin A Bull L Kinzie M Andresen M. Central catheter-associated deep vein thrombosis in cancer: clinical course, prophylaxis, treatment. BMJ Support Palliat Care. (2021) 11(4):371–80. 10.1136/bmjspcare-2019-002106 34413028 PMC8606430

[3] Debourdeau P Farge D Beckers M Baglin C Bauersachs RM Brenner B. International clinical practice guidelines for the treatment and prophylaxis of thrombosis associated with central venous catheters in patients with cancer. J Thromb Haemostasis. (2013) 11(1):71–80. 10.1111/jth.12071 23217208

[4] Cheng F Fu Q He P. Chinese expert consensus on prevention and treatment catheter related venous thrombosis (2020 edition). Chin J Prac Surg. (2020) 40(4):377–83.

[5] Wang P He L Yuan Q Lu J Ji Q Peng A. Risk factors for peripherally inserted central catheter-related venous thrombosis in adult patients with cancer. Thromb J. (2024) 22(1):6. 10.1186/s12959-023-00574-4 38178101 PMC10768137

[6] Nie S Wang L Ma S Sun H. Trends in the prevalence and risk factors for peripherally inserted central catheter-related complications in cancer patients from 2016 to 2022: a multicenter study. Support Care Cancer. (2024) 32(4):239. 10.1007/s00520-024-08444-z 38512390

[7] Chen X-F Wu H-J Li T Liu J-B Zhou W-J Guo Q. Establishment and validation of a predictive model for peripherally inserted central catheter-related thrombosis in patients with liver cancer. World J Gastrointest Surg. (2024) 16(7):2221–31. 10.4240/wjgs.v16.i7.2221 39087116 PMC11287695

[8] Chen Y Chen H Yang J Jin W Fu D Liu M. Patterns and risk factors of peripherally inserted central venous catheter-related symptomatic thrombosis events in patients with malignant tumors receiving chemotherapy. J Vasc Surg Venous Lymphat Disord. (2020) 8(6):919–29. 10.1016/j.jvsv.2020.01.010 32205131

[9] Onyeama SJN Hanson SJ Dasgupta M Baker K Simpson PM Punzalan RC. Central venous catheter–associated venous thromboembolism in children with hematologic malignancy. J Pediatr Hematol Oncol. (2018) 40(8):e519–e24. 10.1097/MPH.0000000000001229 29863581

[10] Hu J Xu B Yao N Peng S Lv J Yu H. Risk factors of catheter-related thrombosis in elderly patients with lung cancer based on thromboelastography: a retrospective, case-control study. Semin Thromb Hemost. (2025) 52(04):476–86. 10.1055/a-2576-4895 40315871

[11] Fu J Cai W Zeng B He L Bao L Lin Z. Development and validation of a predictive model for peripherally inserted central catheter-related thrombosis in breast cancer patients based on artificial neural network: a prospective cohort study. Int J Nurs Stud. (2022) 135:104341. 10.1016/j.ijnurstu.2022.104341 36084529

[12] Ma G Chen S Peng S Yao N Hu J Xu L. Construction and validation of a nomogram prediction model for the catheter-related thrombosis risk of central venous access devices in patients with cancer: a prospective machine learning study. J Thromb Thrombolysis. (2024) 58(2):220–31. 10.1007/s11239-024-03045-3 39363143

[13] Ellis ML Okano S McCann A McDowall A Van Kuilenburg R McCarthy AL. Catheter-related thrombosis incidence and risk factors in adult cancer patients with central venous access devices. Intern Med J. (2020) 50(12):1475–82. 10.1111/imj.14780 32043739

[14] Piran S Ngo V McDiarmid S L Gal G Petrcich W Carrier M. Incidence and risk factors of symptomatic venous thromboembolism related to implanted ports in cancer patients. Thromb Res. (2014) 133(1):30–3. 10.1016/j.thromres.2013.10.026 24188799

[15] Liu B Xie J Sun X Wang Y Yuan Z Liu X. Development and validation of a new clinical prediction model of catheter-related thrombosis based on vascular ultrasound diagnosis in cancer patients. Front Cardiovasc Med. (2020) 7:571227. 10.3389/fcvm.2020.571227 33195460 PMC7649194

[16] Lipe DN Qdaisat A Rajha E Al-Breiki A Cruz Carreras MT Chaftari P. Characteristics and predictors of venous thrombosis recurrence in patients with cancer and catheter-related thrombosis. Res Pract Thromb Haemost. (2022) 6(6):e12761. 10.1002/rth2.12761 36000091 PMC9391569

[17] Wang G Wang H Shen Y Dong J Wang X Wang X. Association between ABO blood group and venous thrombosis related to the peripherally inserted central catheters in cancer patients. J Vasc Access. (2020) 22(4):590–6. 10.1177/1129729820954721 32880203

[18] Sun C-J Ma L Wang S-Y Xue Q-Y. A quasi-experimental study on the preventive effect of risk-stratified nursing interventions for PICC-related thrombosis in cancer patients. Risk Manag Healthc Policy. (2025) 18:3209–20. 10.2147/RMHP.S521571 41030669 PMC12477278

[19] Hoppe A Rupa-Matysek J Małecki B Dytfeld D Hoppe K Gil L. Risk factors for catheter-related thrombosis in multiple myeloma patients undergoing autologous stem cell transplantation. Medicina (B Aires). (2021) 57(10):1020. 10.3390/medicina57101020 PMC8537595 34684057

[20] Revel-Vilk S Yacobovich J Tamary H Goldstein G Nemet S Weintraub M. Risk factors for central venous catheter thrombotic complications in children and adolescents with cancer. Cancer. (2010) 116(17):4197–205. 10.1002/cncr.25199 20533566

[21] Aw A Carrier M Koczerginski J McDiarmid S Tay J. Incidence and predictive factors of symptomatic thrombosis related to peripherally inserted central catheters in chemotherapy patients. Thromb Res. (2012) 130(3):323–6. 10.1016/j.thromres.2012.02.048 22444157

[22] Yi X- Chen J Li J Feng L Wang Y Zhu J-A. Risk factors associated with PICC-related upper extremity venous thrombosis in cancer patients. J Clin Nurs. (2013) 23(5-6):837–43. 10.1111/jocn.12227 23710585

[23] Peng S-Y Wei T Li X-Y Yuan Z Lin Q. A model to assess the risk of peripherally inserted central venous catheter-related thrombosis in patients with breast cancer: a retrospective cohort study. Support Care Cancer. (2021) 30(2):1127–37. 10.1007/s00520-021-06511-3 34432170

[24] Liang Y-J He Y Li J-M Chen L-M Chen L-P Wang C. The incidence and predictors of symptomatic venous thromboembolism associated with peripherally inserted central catheters in patients with nasopharyngeal carcinoma. Onco Targets Ther. (2018) 11:3119–27. 10.2147/OTT.S164723 29872323 PMC5975597

[25] Wang X-X He Y Chu J Xu J-S. Risk factors analysis and the establishment of nomogram prediction model for PICC-related venous thrombosis in patients with lymphoma: a double-center cohort-based case-control study. Front Oncol. (2024) 14:1347297. 10.3389/fonc.2024.1347297 38559558 PMC10979641

[26] Luo Z Xu W Guo Z Xu M Lv X Chen C. Incidence and risk of catheter-related thrombosis in breast cancer patients with implanted ports. J Infus Nurs. (2025) 48(2):114–22. 10.1097/NAN.0000000000000582 39964872

[27] Debourdeau P Espié M Chevret S Gligorov J Elias A Dupré PF. Incidence, risk factors, and outcomes of central venous catheter-related thromboembolism in breast cancer patients: the CAVECCAS study. Cancer Med. (2017) 6(11):2732–44. 10.1002/cam4.1201 28980454 PMC5673948

[28] Tan L Sun Y Zhu L Lei X Liang D Rao N. Risk factors of catheter-related thrombosis in early-stage breast cancer patients: a single-center retrospective study. Cancer Manag Res. (2019) 11:8379–89. 10.2147/CMAR.S212375 31571994 PMC6750851

[29] Wang G-D Wang H-Z Shen Y-F Dong J Wang X-P Wang X-Z. The influence of venous characteristics on peripherally inserted central catheter-related symptomatic venous thrombosis in cancer patients. Cancer Manag Res. (2020) 12:11909–20. 10.2147/CMAR.S282370 33244268 PMC7685368

[30] Kato F Takeuchi H Matsuda S Kawakubo H Omori T Kitagawa Y. Incidence of and risk factors for venous thromboembolism during surgical treatment for esophageal cancer: a single-institution study. Surg Today. (2015) 46(4):445–52. 10.1007/s00595-015-1196-1 26094966

[31] Lee AYY Levine MN Butler G Webb C Costantini L Gu C. Incidence, risk factors, and outcomes of catheter-related thrombosis in adult patients with cancer. J Clin Oncol. (2006) 24(9):1404–8. 10.1200/JCO.2005.03.5600 16549834

[32] Wei T Peng S- Li X- Yuan Z Lin Q. Upper limb lymphedema impacts the risk of peripherally inserted central catheter-related thrombosis in patients with breast cancer. Lymphology. (2022) 55:178–87. 10.2458/lymph.5739 37553006

[33] Chen P Wan G Zhu B. Incidence and risk factors of symptomatic thrombosis related to peripherally inserted central catheter in patients with lung cancer. J Adv Nurs. (2020) 77(3):1284–92. 10.1111/jan.14666 33249623

[34] Lin S Zhu N Yihan Zhang Du L Zhang S. Development and validation of a prediction model of catheter-related thrombosis in patients with cancer undergoing chemotherapy based on ultrasonography results and clinical information. J Thromb Thrombolysis. (2022) 54(3):480–91. 10.1007/s11239-022-02693-7 35972592 PMC9553810

[35] Song X Lu H Chen F Bao Z Li S Li S. A longitudinal observational retrospective study on risk factors and predictive model of PICC associated thrombosis in cancer patients. Sci Rep. (2020) 10(1):10090. 10.1038/s41598-020-67038-x 32572092 PMC7308336

[36] Yue J Zhang Y Xu F Mi A Zhou Q Chen B. A clinical study of peripherally inserted central catheter-related venous thromboembolism in patients with hematological malignancies. Sci Rep. (2022) 12(1):9871. 10.1038/s41598-022-13916-5 35701467 PMC9197841

[37] Feng Y Zheng R Fu Y Xiang Q Yue Z Li J. Assessing the thrombosis risk of peripherally inserted central catheters in cancer patients using Caprini risk assessment model: a prospective cohort study. Support Care Cancer. (2021) 29(9):5047–55. 10.1007/s00520-021-06073-4 33594508

[38] De Cicco M Matovic M Balestreri L De Angelis V Fracasso A Morassut S. Antithrombin III deficiency as a risk factor for catheter-related central vein thrombosis in cancer patients. Thromb Res. (1995) 78(2):127–37. 10.1016/0049-3848(95)00041-0 7482430

[39] Mandalà M Curigliano G Bucciarelli P Ferretti G Mannucci PM Colleoni M. Factor V Leiden and G20210A prothrombin mutation and the risk of subclavian vein thrombosis in patients with breast cancer and a central venous catheter. Ann Oncol. (2004) 15(4):590–3. 10.1093/annonc/mdh146 15033664

[40] Wang G Li Y Wu C Guo L Hao L Liao H. The clinical features and related factors of PICC-related upper extremity asymptomatic venous thrombosis in cancer patients. Medicine (Baltimore). (2020) 99(12):e19409. 10.1097/MD.0000000000019409 32195936 PMC7220136

[41] Li X Wang G Yan K Yin S Wang H Wang Y. The incidence, risk factors, and patterns of peripherally inserted central catheter-related venous thrombosis in cancer patients followed up by ultrasound. Cancer Manag Res. (2021) 13:4329–40. 10.2147/CMAR.S301458 34103988 PMC8179741

[42] Lan Y Wu L Guo J Wang J Guan H Li B. Risk factors for totally implantable access ports-associated thrombosis in pediatric oncology patients. Sci Rep. (2023) 13(1):3553. 10.1038/s41598-023-30763-0 36864082 PMC9981621

[43] Gao Y Liu Y Chen W Wei L Song L Ma X. Peripherally inserted central catheter thrombosis incidence and risk factors in cancer patients: a double-center prospective investigation. Ther Clin Risk Manag. (2015) 11:153–60. 10.2147/TCRM.S73379 25673995 PMC4321638

[44] Haggstrom L Parmar G Brungs D. Central venous catheter thrombosis in cancer: a multi-centre retrospective study investigating risk factors and contemporary trends in management. Clinical Medicine Insights: Oncology. (2020) 14:1179554920953097. 10.1177/1179554920953097 32943968 PMC7466879

[45] Al-Asadi O Almusarhed M Eldeeb H. Predictive risk factors of venous thromboembolism (VTE) associated with peripherally inserted central catheters (PICC) in ambulant solid cancer patients: retrospective single centre cohort study. Thromb J. (2019) 17(1):2. 10.1186/s12959-019-0191-y 30697126 PMC6346522

[46] Qiu X- Guo Y Fan H- Shao J Zhang X. Incidence, risk factors and clinical outcomes of peripherally inserted central catheter spontaneous dislodgment in oncology patients: a prospective cohort study. Int J Nurs Stud. (2014) 51(7):955–63. 10.1016/j.ijnurstu.2013.10.021 24246096

[47] Feng Y Fu Y Xiang Q Xie L Yu C Li J. Plasminogen activator inhibitor-1 gene promoter 4G/5G polymorphism and risks of peripherally inserted central catheter-related venous thrombosis in patients with lung cancer: a prospective cohort study. Support Care Cancer. (2021) 29(11):6431–9. 10.1007/s00520-021-06207-8 33893843

[48] Verso M Agnelli G Kamphuisen PW Ageno W Bazzan M Lazzaro A. Risk factors for upper limb deep vein thrombosis associated with the use of central vein catheter in cancer patients. Intern Emerg Med. (2008) 3(2):117–22. 10.1007/s11739-008-0125-3 18317868

[49] Gould JR Carloss HW Skinner WL. Groshong catheter-associated subclavian venous thrombosis. Am J Med. (1993) 95:419–23. 10.1016/0002-9343(93)90312-D 8213875

[50] De Cicco M Matovic M Balestreri L Panarello G Fantin D Morassut S. Central venous thrombosis: an early and frequent complication in cancer patients bearing long-term silastic catheter. A prospective study. Thromb Res. (1997) 86(2):101–13. 10.1016/S0049-3848(97)00054-6 9175232

[51] Puel V Caudry M Le Métayer P Baste J-C Midy D Marsault C. Superior vena cava thrombosis related to catheter malposition in cancer chemotherapy given through implanted ports. Cancer. (1993) 72(7):2248–52. 10.1002/1097-0142(19931001)72:7<2248::AID-CNCR2820720731>3.0.CO;2-U 8374884 10.1002/1097-0142(19931001)72:7<2248::aid-cncr2820720731>3.0.co;2-u

[52] Kang J Sun W Li H Ma E Wang K Chen W. Peripherally inserted central catheter-related vein thrombosis in breast cancer patients. J Vasc Access. (2015) 17(1):67–71. 10.5301/jva.5000457 26349890

[53] Wang G Shen Y Wang X Dong J Wang X Zheng Y. Impact of vascular morphology and body mass index on PICC-related thrombosis in cancer patients: a prospective cohort study. Int J Surg. (2026) 112(3):6837–47. 10.1097/JS9.0000000000004130

[54] Decousus H Bourmaud A Fournel P Bertoletti L Labruyère C Presles E. Cancer-associated thrombosis in patients with implanted ports: a prospective multicenter French cohort study (ONCOCIP). Blood. (2018) 132(7):707–16. 10.1182/blood-2018-03-837153 29980524

[55] Chen G Lin A Shen R Ge J Zhong T You G. Incidence and characteristics of catheter-related thrombosis in children with malignant tumors: a single-center retrospective cohort study. BMC Pediatr. (2025) 25(1):970. 10.1186/s12887-025-06334-9 41316114 PMC12664246

[56] Refaei M Fernandes B Brandwein J Goodyear MD Pokhrel A Wu C. Incidence of catheter-related thrombosis in acute leukemia patients: a comparative, retrospective study of the safety of peripherally inserted vs. Centrally inserted central venous catheters. Ann Hematol. (2016) 95(12):2057–64. 10.1007/s00277-016-2798-4 27542955

[57] Noailly Charny P-A Bleyzac N Ohannessian R Aubert E Bertrand Y Renard C. Increased risk of thrombosis associated with peripherally inserted central catheters compared with conventional central venous catheters in children with leukemia. J Pediatr. (2018) 198:46–52. 10.1016/j.jpeds.2018.03.026 29709343

[58] Chopra V Ratz D Kuhn L Lopus T Lee A Krein S. Peripherally inserted central catheter-related deep vein thrombosis: contemporary patterns and predictors. J Thromb Haemostasis. (2014) 12(6):847–54. 10.1111/jth.12549 24612469

[59] Ahn DH Illum HB Wang DH Sharma A Dowell JE. Upper extremity venous thrombosis in patients with cancer with peripherally inserted central venous catheters: a retrospective analysis of risk factors. Journal of Oncology Practice. (2013) 9(1):e8–e12. 10.1200/JOP.2012.000595 23633980 PMC3545673

[60] Hu Z Luo M He R Wu Z Fan Y Li J. Development and validation of a risk prediction model for PICC-related venous thrombosis in patients with cancer: a prospective cohort study. Sci Rep. (2025) 15(1):4654. 10.1038/s41598-025-89260-1 39920370 PMC11805956

[61] Wang X Dai X He Y Chu J Wang Y. Establishment and validation of a predictive nomogram for central venous catheter-related thrombosis in cancer patients: a retrospective nested case-control study. Front Oncol. (2024) 14:1418273. 10.3389/fonc.2024.1418273 39220644 PMC11361954

[62] Weng G Wu X Zheng S. Influence factors analyses of PICC-related bloodstream infection, PICC-related venous thrombosis, and infected puncture site and their influence on cancer patients' death: a retrospective cross-sectional study. Cancer Med. (2025) 14(7):e70841. 10.1002/cam4.70841 40171872 PMC11962649

