# Supplementary material for: Risk factors for catheter-related thrombosis in cancer patients: a systematic review
Source: Front Cardiovasc Med. 2026 Sep 18;13:1914685. doi: 10.3389/fcvm.2026.1914685 (PMC13631437; doi:10.3389/fcvm.2026.1914685)
Supplement: Supplementary file 1 [file Datasheet1.pdf]

Supplementary Table S1 . Multivariate analysis: characteristics of included studies

| Author &year | Study population | Sample size | Diagnostic Tools/Diagnostic Methods | Factors | OR/HR [95%CI] | p |
|---|---|---|---|---|---|---|
| Pinghu Wang 2024 (Pinghu Wang et al., 2024) | Adult patients with cancer who received PICC insertion | 2227 | color Doppler ultrasound (CDU) | Age > 65 years old | 1.791[1.343–2.389] | <0.001 |
| | | | | Male | 1.398[1.057–1.849] | 0.019 |
| | | | | White blood cell count > 9.5 × 109 /L | 1.422[1.041–1.942] | 0.027 |
| | | | | APTT < 25 s | 2.006[1.431–2.811] | <0.001 |
| | | | | Gastrointestinal Tumor | 2.191[1.406–3.414] | 0.001 |
| | | | | Any infection | 7.619[5.783–10.037] | <0.001 |
| | | | | Cisplatin | 2.347[1.714–3.214] | <0.001 |
| | | | | Vincristine | 2.329[1.447–3.749] | <0.001 |
| | | | | Polyurethane PICC | 2.449[1.863–3.219] | <0.001 |
| | | | | Open-ended catheter | 1.660[1.131–2.439] | 0.010 |
| | | | | Keeping time of the catheter (days) | 1.003[1.001–1.005] | 0.005 |

PICC = Peripherally Inserted Central Catheter；CDU = Color Doppler Ultrasound；APTT = Activated Partial Thromboplastin Time；OR = Odds Ratio；HR = Hazard Ratio；CI = Confidence Interval

Supplementary Table S1 continue. Multivariate analysis: characteristics of included studies

| Author &year | Study population | Sample size | Diagnostic Tools/Diagnostic Methods | Factors | OR/HR [95%CI] | p |
|---|---|---|---|---|---|---|
| Shengxiao Nie 2024 (Shengxiao Nie et al., 2024) | Cancer patients with PICC | 3573 | Ultrasound color ultrasound Doppler | Hospital level (tertiary vs secondary) | 1.756[1.386-2.225] | <0.001 |
| | | | | Hospital nature (specialized vs comprehensive) | 3.449[2.292-5.190] | <0.001 |
| | | | | Gender (men vs women) | 2.331[1.854-2.931] | <0.001 |
| | | | | Age (≥65 years vs <65 years) | 1.468[1.165-1.849] | 0.001 |
| | | | | Diagnosis (lung cancer vs breast cancer) | 2.384[1.756-3.236] | <0.001 |
| | | | | Diagnosis (head and neck cancer vs breast cancer) | 5.042[2.584-9.837] | <0.001 |
| | | | | Diagnosis (cancer of the digestive system vs breast cancer) | 2.445[1.805-3.312] | <0.001 |
| | | | | Diagnosis (cancer of the reproductive system vs breast cancer) | 2.424[1.604-3.663 | <0.001 |
| | | | | Diagnosis (cancer of the hematological system vs breast cancer) | 3.975[2.578-6.130] | <0.001 |
| | | | | Diagnosis (other cancer vs breast cancer) | 3.872[2.316-6.471] | <0.001 |
| | | | | History of deep vein catheterization (yes vs no) | 2.933[2.073-4.150] | <0.001 |
| | | | | Chemotherapy drug administration (yes vs no) | 53.183[20.389-138.724] | <0.001 |
| | | | | Type of PICC (open-ended vs tridirectional valve) | 1.789[1.224-2.617] | 0.003 |

PICC = Peripherally Inserted Central Catheter； OR = Odds Ratio； HR = Hazard Ratio； CI = Confidence Interval

Supplementary Table S1 continue. Multivariate analysis: characteristics of included studies

| Author &year | Study population | Sample size | Diagnostic Tools/Diagnostic Methods | Factors | OR/HR [95%CI] | p |
|---|---|---|---|---|---|---|
| Xiao-Fei Chen, 2024 (Xiao-Fei Chen et al., 2024) | hospitalized patients with liver cancer | 255 | Ultrasound Doppler | Age ≥ 60 years | 1.907[1.038-3.491] | 0.036 |
| | | | | KPS score ≤ 50 points | 2.045[1.276-3.899] | 0.038 |
| | | | | Parenteral nutrition | 9.467[2.011-43.596] | 0.005 |
| | | | | Duration of the tube is from 1 to 6 months | 0.506[0.271-0.955] | 0.032 |
| | | | | Tube time > 6 months | 0.185[0.042-0.856] | 0.030 |
| | | | | Phase III to IV | 2.844[1.865-3.621] | 0.032 |
| | | | | Distant metastasis | 2.065[1.131-3.778] | 0.018 |
| | | | | Myelosuppression | 2.082[1.114-3.892] | 0.022 |
| | | | | Disability of self-care ability | 13.926[1.661-116.254] | 0.016 |
| Yanliang Chen 2020 (Yanliang Chen et al., 2020) | patients with malignant tumors receiving chemotherapy | 938 | color Doppler ultrasonography or computed tomography pulmonary angiography | Age >60 years | 2.142 [1.118-4.103] | 0.022 |
| | | | | Chemotherapy regimen containing fluorouracil | 2.429 [1.013-5.825] | 0.047 |
| | | | | Hypertension with medication | 0.306 [0.113-0.828] | 0.020 |

KPS = Karnofsky Performance Status; OR = Odds Ratio; HR = Hazard Ratio; CI = Confidence Intervalstudies

Supplementary Table S1 continue.Multivariate analysis: characteristics of included studies

| Author &year | Study population | Sample size | Diagnostic Tools/Diagnostic Methods | Factors | | OR/HR [95%CI] | p |
|---|---|---|---|---|---|---|---|
| Sara-Jane N 2018 (Sara-Jane N et al., 2018) | Children with hematologic malignancy (<18 years old, newly diagnosed) with central venous catheter (CVC) placement | 198 | Doppler ultrasound | Cancer diagnosis age (older) | | 1.09 [1.02-1.17] | 0.008 |
| | | | | CVC type (PICC) | | 10.52 [2.19-50.59] | 0.003 |
| | | | | Comorbidity (sepsis) | | 0.13 [0.04-0.42] | 0.0007 |
| | | | | tPA use for CVC occlusion | | 0.05 [0.01-0.33] | 0.002 |
| Jiaji Hu, MSc 2025 (Jiaji Hu et al., 2025) | elderly patients with lung cance | 663 | ultrasound | TEG values | TEG parameter-R (min) | 0.849[0.763-0.945] | 0.003 |
| | | | | | TEG parameter-K (min) | 0.571[0.404-0.807] | 0.001 |
| | | | | Coagulation routine:Prothrombin time (PT) | | 0.904[0.830-0.985] | 0.022 |
| | | | | Blood routine | Platelet count (109/L) | 1.006[1.004-1.009] | <0.001 |
| | | | | | Hemoglobin (g/L) | 1.020[1.009-1.031] | <0.001 |
| | | | | Other clinical factors | Age | 1.073[1.033-1.113] | <0.001 |
| | | | | | Sex:Male | 4.534[2.815-7.304] | <0.001 |
| | | | | | Physical activity disorder-Yes | 9.038[4.462-18.309] | <0.001 |
| | | | | | Surgery 1 month-Yes | 2.258[1.420-3.590] | 0.001 |
| | | | | | Diabetes-Yes | 2.478[1.373-4.472] | 0.003 |
| | | | | | Hypertension-Yes | 2.386[1.505-3.784] | <0.001 |

CVC = Central Venous Catheter；PICC = Peripherally Inserted Central Catheter；tPA = Tissue Plasminogen Activator；TEG = Thromboelastography；PT = Prothrombin Time；

OR = Odds Ratio； HR = Hazard Ratio； CI = Confidence Interval

Supplementary Table S1 continue.Multivariate analysis: characteristics of included studies

| Author &year | Study population | Sample size | Diagnostic Tools/Diagnostic Methods | Factors | OR/HR [95%CI] | p |
|---|---|---|---|---|---|---|
| Jianqin Fu 2022 (Jianqin Fu et al., 2022) | Adult female patients with pathologically confirmed breast cancer | 1844 | Color Doppler ultrasound | Insertion attempts (≥2 vs 1) | 9.457 [3.144-28.448] | <0.001 |
| | | | | Upper extremity activity (poor vs ideal) | 3.628 [2.309-5.702] | <0.001 |
| | | | | CVC history (yes vs no) | 2.799 [1.506-5.202] | 0.001 |
| | | | | Comorbidities (yes vs no) | 1.709 [1.161-2.515] | 0.007 |
| | | | | Age (per 1-year increase) | 1.028 [1.010-1.046] | 0.002 |
| Guiyuan Ma 2024 (Guiyuan Ma et al., 2024) | patients with cancer with CVADs placed | 1500 | Doppler ultrasound | Age | 1.686[1.313–2.166] | <0.001 |
| | | | | Catheterisation method | 0.364[0.224–0.592] | <0.001 |
| | | | | Catheter value | 0.478[0.325–0.702] | <0.001 |
| | | | | Catheter material | 0.759[0.603–0.955] | 0.019 |
| | | | | Infection | 5.700[3.727–8.719] | <0.001 |
| | | | | Catheterisation history | 1.863[1.296–2.677] | 0.001 |
| | | | | D-Dimer concentration | 3.156[2.234–4.459] | <0.001 |
| | | | | Operation history | 8.982[6.134–13.151] | <0.001 |
| | | | | Anaemia | 2.580[1.805–3.688] | <0.001 |
| | | | | Diabetes | 2.116[1.258–3.560] | 0.005 |
| | | | | Targeted drugs | 1.631[1.079–2.464] | 0.020 |
| M. L. Ellis, S 2020 (M. L. Ellis, S et al., 2020) | Adult cancer patients | 3130 | Color Doppler ultrasound | CVAD type: Peripherally Inserted Central Catheter (PICC) (vs Implantable port) | 22.2 [2.9-170.6] * | 0.003 |
| | | | | Prior CRT history | 1.81 [1.19-2.77] * | 0.006 |
| | | | | Age group: 50-61 years (vs <50 years) | 0.57 [0.39-0.84] * | 0.005 |
| | | | | Age group: >61 years (vs <50 years) | 0.63 [0.45-0.89] * | 0.005 |

CVC = Central Venous Catheter; CVAD = Central Venous Access Device; CRT = Catheter-Related Thrombosis;OR = Odds Ratio; HR = Hazard Ratio; CI = Confidence

Interval;*=HR

Supplementary Table S1 continue.Multivariate analysis: characteristics of included studies

| Author &year | Study population | Sample size | Diagnostic Tools/Diagnostic Methods | Factors | | OR/HR [95%CI] | p |
|---|---|---|---|---|---|---|---|
| Siavash Piran 2014 (Siavash Piran et al., 2014) | cancer patients with a newly inserted implanted port for deliverance of chemotherapy | 400 | pulmonary embolism (PE) symptomatic upper Ultrasound | Gender (Male vs. Female) | | 2.17 [1.04–4.55] | 0.0383 |
| | | | | Metastases | | 8.2 [1.92–35.2] | 0.0045 |
| Liu et al./2020 (Liu et al., 2020) | cancer patients who received a catheter and underwent a vascular ultrasound examination. | 3131 | Vascular ultrasound | Sex(male vs.female) | | 1.43[1.06–1.92] | 0.018 |
| | | | | Cancer type(Breast cancer vs.Urogenital cancer) | | 2.82[1.50–5.29] | 0.001 |
| | | | | Cancer type(Thoracic cancer vs.Urogenital cancer) | | 6.25[3.35–11.68] | 0.000 |
| | | | | Cancer type(Gastrointestinal cancers vs.Urogenital cancer) | | 5.80[3.08–10.93] | 0.000 |
| | | | | Cancer type(Hematological cancers vs.Urogenital cancer) | | 5.53[2.46–12.46] | 0.000 |
| | | | | Cancer type(Other tumors vs.Urogenital cancer) | | 3.15[1.09–9.14] | 0.035 |
| Demis N. Lipe MD 2022 (Demis N. Lipe et al., 2022) | Cancer patients with symptomatic upper-extremity deep vein thrombosis | 257 | imaging reports Vascular bed or pulmonary embolism, | Recurrence within 3 months | Catheter removal before anticoagulation | 5.07 [1.53-23.18] | 0.02 |
| | | | | | Catheter reinsertion after removal | 9.96 [1.82-186.75] | 0.03 |
| | | | | | Male gender | 3.57[1.15-13.65] | 0.04 |
| | | | | 1-year venous thromboembolic recurrence | Catheter removal before anticoagulation | 3.47 [1.34-9.01]* | 0.01 |
| | | | | | Catheter reinsertion after removal | 3.07 [1.09-8.67]* | 0.03 |

PE = Pulmonary Embolism； OR = Odds Ratio； HR = Hazard Ratio； CI = Confidence Interva； *=HR

Supplementary Table S1 continue.Multivariate analysis: characteristics of included studies

| Author &year | Study population | Sample size | Diagnostic Tools/Diagnostic Methods | Factors | | OR/HR [95%CI] | p |
|---|---|---|---|---|---|---|---|
| Guodong Wang 2020 (Guodong Wang et al., 2020) | cancer patients | 2315 | a color Doppler ultrasound | Blood group | A | 2.072 [1.204–3.566] | / |
| | | | | | AB | 2.706 [1.432–5.114] | / |
| | | | | | B | 1.944 [1.146–3.298] | / |
| | | | | Prior VTE | | 1.987 [1.020–3.873] | 0.044 |
| Chun-Jie Sun 2025 (Chun-Jie Sun et al., 2025 | all chemotherapy patients scheduled for PICC plac4ement | 236 | Doppler ultrasound | Tumor stage III/IV | | 2.556[1.798-5.564] | 0.031 |
| | | | | History of thrombosis | | 19.273[10.674-37.561 ] | 0.033 |
| | | | | Complicated by diabetes | | 2.572[1.027-5.712] | 0.035 |
| | | | | Number of punctures | | 19.886[8.704-33.492] | 0.040 |
| | | | | Catheter tip position | | 14.339[8.916-29.795] | 0.004 |
| | | | | D-Dimer | | 9.528[6.703-15.597] | 0.036 |
| | | | | Use of anticoagulants | | 0.449[0.089-0.874] | 0.017 |
| Anna Hoppe 2021 (Anna Hoppe et al., 2021) | Adult patients with pathologically confirmed multiple myeloma (MM) | 276 | Doppler ultrasound | Previous thromboembolic event (TE) before ASCT | | 2.78［1.21-6.40］ | 0.016 |
| | | | | CVC-related infection | | 2.88［1.24-6.70］ | 0.014 |
| | | | | Non-infectious complications | | 2.75［1.10-6.91］ | 0.031 |

VTE = Venous Thromboembolism;ASCT = Autologous Stem Cell Transplantation；CVC = Central Venous Catheter；* = HR；OR = Odds Ratio；HR = Hazard Ratio；CI = Confidence Interval

Supplementary Table S1 continue.Multivariate analysis: characteristics of included studies

| Author &year | Study population | Sample size | Diagnostic Tools/Diagnostic Methods | Factors | OR/HR [95%CI] | p |
|---|---|---|---|---|---|---|
| S. Revel-Vilk2010 (S. Revel-Vilk et al., 2010) | Children and Adolescents With Cancer | 262 | suspected by the primary physician and confirmed by diagnostic imaging | Positive family history of thrombosis | 2.16[1.21-3.84]* | 0.007 |
| AwAndrew 2012 (Andrew Aw et al., 2012) | cancer patients obtained PICC lines for the administration of chemotherapy | 340 | Compression ultrasound | Metastatic cancer | 3.34 [1.17–9.51] | 0.024 |
| | | | | Diabetes mellitus | 3.18 [1.06–9.53] | 0.039 |
| Xiao-lei Yi 2013 (Xiao-lei Yi et al., 2013) | Consecutive patients with cancer | 89 | Doppler sonography | Chemotherapy history | 3.19[1.07–9.77] | 0.017 |
| | | | | Less activity | 2.11[0.77–8.10] | 0.009 |
| | | | | Diabetes | 1.12[0.89-4.57] | 0.006 |

PICC = Peripherally Inserted Central Catheter； * = HR； OR = Odds Ratio； HR = Hazard Ratio； CI = Confidence Interval

Supplementary Table S1 continue.Multivariate analysis: characteristics of included studies

| Author &year | Study population | Sample size | Diagnostic Tools/Diagnostic Methods | Factors | OR/HR [95%CI] | p |
|---|---|---|---|---|---|---|
| Si-yi Peng 2021 (Si-yi Peng et al., 2021) | breast cancer patients | 1284 | ultrasonography | COPD(vs. none) | 7.967[1.051–60.367] | 0.045 |
| | | | | PLT (vs. per unit increase) | 3.783[1.756–8.149] | 0.001 |
| | | | | D-dimer (vs. per unit increase) | 3.673[1.698–7.946] | 0.001 |
| | | | | APTT(vs. per unit decrease) | 7.112[1.278–39.571] | 0.025 |
| | | | | Menopause(vs. none) | 2.242[1.015–4.955] | 0.046 |
| | | | | breast operation (vs. none) | 0.338[0.141–0.808] | 0.015 |
| | | | | upper limb lymphedema (vs. none) | 28.040[8.703–90.344] | 0.000 |
| | | | | endocrine therapy (vs. none) | 4.372[1.355–14.105] | 0.014 |
| | | | | CVC placement history (vs. none) | 10.12[1.763-58.081] | 0.009 |
| Yu-Jing Liang 2018 (Yu-Jing Liang et al., 2018) | Nasopharyngeal carcinoma (NPC) patients with peripherally inserted central catheter (PICC) insertion | 1363 | Color Doppler ultrasound | VTE history | 109.772 [12.572-958.468] | <0.001 |
| | | | | ECOG score (worse functional status) | 4.162[1.385-12.513] | 0.011 |
| | | | | M stage (metastatic) | 2.717[1.178-6.266] | 0.019 |
| | | | | BMI (kg/m²) | 0.900[0.834-0.972] | 0.007 |

COPD = Chronic Obstructive Pulmonary Disease； PLT = Platelet； APTT = Activated Partial Thromboplastin Time； CVC = Central Venous Catheter； VTE = Venous Thromboembolism； ECOG = Eastern Cooperative Oncology Group； BMI = Body Mass Index;OR = Odds Ratio； HR = Hazard Ratio； CI = Confidence Interval；

Supplementary Table S1 continue.Multivariate analysis: characteristics of included studies

| Author &year | Study population | Sample size | Diagnostic Tools/Diagnostic Methods | Factors | | OR/HR [95%CI] | p |
|---|---|---|---|---|---|---|---|
| Xue-xing Wang 2024 (Xue-xing Wang et al., 2024) | lymphoma patients | 305 | ultrasound examinatio, intravenous angiography, non-enhanced ultrasound, or enhanced echocardiography | Activity amount-Frequently | | 0.217[0.045-1.038] | 0.006 |
| | | | | Thrombosis history (within the last 12 months) | | 33.733[5.964-190.806 ] | <0.001 |
| | | | | ATIII>97.5 | | 0.182[0.052-0.634] | 0.007 |
| | | | | Total cholesterol (mmol/L)≥5.36 | | 3.865[1.087-13.744] | 0.037 |
| | | | | Position of catheter tip n (%) | | 0.057[0.012-0.282] | <0.001 |
| | | | | D-Dimer (mg/L)>0.55 | | 5.765[1.205-25.578] | 0.028 |
| Zebing Luo, MSN 2025 (Zebing Luo et al., 2025) | breast cancer | 461 | imaging | Asymptomatic Thrombosis (%) | Number of comorbidities:0 | 6.642[2.379-18.544] | <0.001 |
| | | | | | Number of comorbidities: 1 | 8.927 [3.155-25.257] | <0.001 |
| | | | | Symptomatic Thrombosis (%) | Chemotherapy | 0.246 [0.135-0.446] | <0.001 |
| | | | | | Base material of TIVAP reservoir: Ceramics | 2.707 [1.263-5.803] | 0.010 |
| | | | | | Site of catheter: Left | 0.330 [0.143-0.761] | 0.009 |
| | | | | | Clinical stage: III | 0.043 [0.003-0.733] | 0.030 |
| | | | | | Chemotherapy | 0.086 [0.009-0.855] | 0.036 |

ATIII = Antithrombin III； TIVAP = Totally Implantable Venous Access Port； OR = Odds Ratio； HR = Hazard Ratio； CI = Confidence Interval

Supplementary Table S1 continue.Multivariate analysis: characteristics of included studies

| Author &year | Study population | Sample size | Diagnostic Tools/Diagnostic Methods | Factors | | OR/HR [95%CI] | p |
|---|---|---|---|---|---|---|---|
| Philippe Debourdeau 2017 (Philippe Debourdeau et al., 2017) | Non-metastatic invasive breast cancer patients | 524 | Doppler ultrasound | All CRT: BMI ≥30 kg/m² | | 2.66 [1.46-4.84] | 0.001 |
| | | | | All CRT: Lobular carcinoma histology | | 2.56 [1.32-4.96] | 0.005 |
| | | | | Symptomatic CRT: Comorbidities ≥1 | | 3.63 [1.24-10.7] | 0.019 |
| | | | | Asymptomatic CRT: BMI ≥30 kg/m² | | 2.18 [1.11-4.26] | 0.023 |
| Luyuan Tan 2019 (Luyuan Tan et al., 2019) | Early-stage breast cancer patients | 209 | Ultrasound | Tumor size >2 cm | | 2.735[1.042-7.177] | 0.032 |
| | | | | HBsAg positive (hepatitis B virus infection) | | 2.80[1.03-7.86] | 0.047 |
| | | | | Baseline LDL >3.6 mmol/L | | 2.360[1.06-5.35] | 0.040 |
| Guo-Dong Wang 2020 (Guo-Dong Wang et al., 2020) | Cancer Patients | 2933 | ultrasound | Triglycerides | >1.7mmol/L | 2.279 [1.161–4.475] | 0.017 |
| | | | | Tumor category | Lung cancer | 2.312 [0.622–8.597] | 0.018 |
| | | | | | Tumor of digestive system | 0.426 [0.122–1.484] | |
| | | | | | Urologic tumor | 0.498 [0.068–3.694] | |
| | | | | | Breast cancer | 3.115 [1.402–6.921] | |
| | | | | | Gynecological tumors | 4.406 [0.909–21.36] | |
| | | | | | Others | 0.587 [0.076–4.557] | |
| | | | | Vascular diameter (cm) | 0.35–0.4 | 0.288 [0.057–1.458] | 0.010 |
| | | | | | 0.4–0.46 | 2.435 [0.949–6.247] | |
| | | | | | >0.46 | 1.171 [0.403–3.404] | |
| | | | | Arm circumference | | 1.151 [1.002–1.322] | 0.046 |

CRT = Catheter-Related Thrombosis； BMI = Body Mass Index； HBsAg = Hepatitis B Surface Antigen； LDL = Low-Density Lipoprotein；

OR = Odds Ratio； HR = Hazard Ratio； CI = Confidence Interval

Supplementary Table S1 continue.Multivariate analysis: characteristics of included studies

| Author &year | Study population | Sample size | Diagnostic Tools/Diagnostic Methods | Factors | OR/HR [95%CI] | p |
|---|---|---|---|---|---|---|
| Fumihiko Kato 2015 (Fumihiko Kato et al., 2015) | patients undergoing treatment with curative intent; esophageal cancer | 153 | computed tomography (CT) venous ultrasound scanning | Adenocarcinoma histology | 7.87 [1.50–41.26]* | 0.015 |
| | | | | Neck lymph node dissection | 4.93 [1.33–18.21]* | 0.017 |

CT:computed tomography; OR = Odds Ratio; HR = Hazard Ratio; CI = Confidence Interval; CT = Computed Tomography; * = HR

Supplementary Table S1 continue.Multivariate analysis: characteristics of included studies

| Author &year | Study population | Sample size | Diagnostic Tools/Diagnostic Methods | Factors | OR/HR [95%CI] | p |
|---|---|---|---|---|---|---|
| Agnes Y.Y. Lee 2006 (Agnes Y.Y. Lee et al., 2006) | cancer patients | 444 | Ultrasonography<br>Venography<br>Contrast computed tomography<br>Magnetic resonance imaging<br>Ventilation-perfusion lung scan<br>Spiral CT<br>Pulmonary angiography | More than one insertion attempt | 5.5 [1.2–24.6] | 0.03 |
| | | | | Ovarian cancer | 4.8 [1.5–15.1] | 0.01 |
| | | | | Previous CVC insertion | 3.8 [1.4–10.4] | 0.01 |
| | | | | Catheter blockage | 14.7 [5.5–40] | <0.001 |
| T.Wei 2022 (T. Wei et al., 2022) | breast cancer patients | 1262 | color Doppler ultrasound. | CVC placement history | 5.367 [1.700-16.947]* | 0.004 |
| | | | | Surgery on breast | 0.317[0.170-0.594]* | <0.001 |
| | | | | PLT (higher level, 10^9/L) | 3.274[1.726-6.210]* | <0.001 |
| | | | | D-dimer(above 0.55mg/L) | 3.428[1.909-6.154]* | <0.001 |
| | | | | Limb lymphedema | 12.128[5.551-26.501]* | <0.001 |
| Pei Chen 2020 (Pei Chen et al., 2020) | Adult patients with lung cancer | 748 | Doppler ultrasonography | History of smoking | 2.487 [1.133-5.460] | 0.023 |
| | | | | Chemotherapy agent: Carboplatin | 2.226 [1.189-4.167] | 0.012 |
| | | | | Chemotherapy agent: Docetaxel | 7.227 [1.655-31.562] | 0.009 |
| | | | | PICC size: 5-Fr vs 4-Fr | 3.524 [1.775-6.996] | <0.001 |
| | | | | D-dimer level >0.55 mg/L | 5.321 [2.394-11.826] | <0.001 |

CVC = Central Venous Catheter; CT = Computed Tomography; PLT = Platelet; PICC = Peripherally Inserted Central Catheter; Fr = French; * = HR; OR = Odds Ratio; HR = Hazard Ratio; CI = Confidence Interval

Supplementary Table S1 continue.Multivariate analysis: characteristics of included studies

| Author &year | Study population | Sample size | Diagnostic Tools/Diagnostic Methods | Factors | OR/HR [95%CI] | p |
|---|---|---|---|---|---|---|
| Shanhong Lin 2022 (Shanhong Lin et al., 2022) | Adult patients with pathologically confirmed active cancer | 647 | Vascular ultrasound | Body mass index(BMI) | 1.163 [1.083-1.249] | <0.001 |
| | | | | Cancer thrombosis risk(High vs Low or intermediate) | 3.655 | <0.001 |
| | | | | Cancer thrombosis risk(Very high vs Low or intermediate) | 7.263 [3.421-15.418] | <0.001 |
| | | | | d-dimer level | 3.180 [1.839-5.499] | <0.001 |
| | | | | Blood flow velocity | 0.963 [0.937-0.990] | 0.008 |
| Xiaomin Song 2020 (Xiaomin Song et al., 2020) | Cancer patients | 339 | ultrasound | Performance Status | 2.91 [1.978-4.274] * | <0.001 |
| | | | | Number of puncture attempts | 2.38 [1.619-3.512] * | <0.001 |
| | | | | D-dimer level | 1.12 [1.045-1.200] * | <0.001 |
| | | | | Height | 0.95 [0.911-0.997] * | 0.040 |
| Jing Yue 2022 (Jing Yue et al., 2022)36 | Adult patients with pathologically confirmed hematological malignancies | 117 | Vascular ultrasound | Body Mass Index (BMI) | 1.315 [1.033-1.674] | 0.026 |
| | | | | Prothrombin Time (PT, shortened) | 0.279 [0.097-0.802] | 0.018 |

BMI = Body Mass Index; PT = Prothrombin Time; * = HR; OR = Odds Ratio; HR = Hazard Ratio; CI = Confidence Interval

Supplementary Table S1 continue.Multivariate analysis: characteristics of included studies

| Author &year | Study population | Sample size | Diagnostic Tools/Diagnostic Methods | Factors | | OR/HR [95%CI] | p |
|---|---|---|---|---|---|---|---|
| Rujun Zheng et al.,2021 | Chinese cancer patients with PICCs | 468 | Caprini risk assessment model color Doppler ultrasonography | History of DVT | | 4.475 (1.992-10.055) | <0.001 |
| | | | | Placement attempts >1 | | 5.244 (2.434-11.298) | <0.001 |
| | | | | History of chemotherapy | | 2.014 (1.038-3.910) | 0.038 |
| | | | | Decreased PT | | 3.898 (1.265-12.013) | 0.018 |
| | | | | Male gender | | 0.520 (0.259-1.042) | 0.065 |
| | | | | Caprini score 6 and7 vs score5 | | 2.089 (1.165-3.743) | 0.012 |
| | | | | Caprini score ≥8 vs score5 | | 7.156 (3.157-16.217) | <0.001 |
| M.De Cicco N. 1995 (M.De Cicco et al., 1995) | cancer patients | 40 | peripheral phlebography<br>a quantitative scale<br>laboratory tests<br>pulmonary angiogram and Doppler-mode sonography | Antithrombin III (AT III) deficiency | AT III levels between the two groups | / | <0.001 |
| | | | | | parietal thrombus degree | / | <0.05 |
| | | | | | Overall thrombus degree | / | <0.02 |
| M. Mandalà 2004 (N. Mandalà et al., 2004) | women with locally advanced or metastatic breast cancer | 300 | color Doppler ultrasonography | heterozygous carriers of G1691A factor V | | 6.1 [ 1.1–34.3]. | 0.004 |

PICC = Peripherally Inserted Central Catheter；DVT = Deep Vein Thrombosis；PT = Prothrombin Time；AT III = Antithrombin III；OR = Odds Ratio；HR = Hazard Ratio；CI = Confidence Interval

Supplementary Table S1 continue.Multivariate analysis: characteristics of included studies

| Author &year | Study population | Sample size | Diagnostic Tools/Diagnostic Methods | Factors | OR/HR [95%CI] | p |
|---|---|---|---|---|---|---|
| Guorong Wang 2020 (Guorong Wang et al., 2020) | Adult cancer patients | 127 | Color Doppler ultrasound | Eastern Cooperative Oncology Group (ECOG) score | 2.791 [0.08-0.76]* | 0.000 |
| | | | | Blood flow velocity trend: upward trend (vs downward trend) | 0.250 [2.01-3.87]* | 0.014 |
| Xiang Li 2021 (Xiang Li et al., 2021) | cancer patients | 2353 | Color Doppler ultrasound | ABO blood group (A vs O) | 1.680 [1.009-2.798] | 0.046 |
| | | | | ABO blood group (B vs O) | 1.835 [1.137-2.961] | 0.013 |
| | | | | ABO blood group (AB vs O) | 3.275 [1.840-5.829] | <0.001 |
| | | | | Ratio of PICC diameter:vein diameter >0.35 | 1.689 [1.023-2.789] | 0.041 |
| | | | | High triglyceride level (≥1.7 mmol/L) | 1.561 [1.096-2.223] | 0.014 |
| Yingxia Lan 2023 (Yingxia Lan et al., 2023) | pediatric oncology patient | 587 | Ultrasound imaging | Platelet count | 1.002[1.001-1.003] | 0.004 |
| | | | | the vertical distance from the highest point of the catheter to the upper border of the left and right extremitas sternalis claviculae | 1.031[1.001-1.062] | 0.044 |

ECOG = Eastern Cooperative Oncology Group; * = HR; OR = Odds Ratio; HR = Hazard Ratio; CI = Confidence Interval; PICC = Peripherally Inserted Central Catheter

Supplementary Table S1 continue.Multivariate analysis: characteristics of included studies

| Author &year | Study population | Sample size | Diagnostic Tools/Diagnostic Methods | Factors | OR/HR [95%CI] | p |
|---|---|---|---|---|---|---|
| Yuxiu Liu 2015 (Yuxiu Liu et al., 2015) | cancer patients | 311 | Doppler US | less activity | 9.583[3.171–28.962] | 0.000[a] |
| | | | | Obesity | 3.466[1.290–9.311] | 0.014[b] |
| Lucy Haggstrom 2020 (Lucy Haggstrom et al., 2020) | Adult cancer patients | 317 | Univariable/multivariate Cox proportional hazards regression<br>Chi-square test<br>Independent samples t-test<br>SPSS 25.0<br>Imaging | CVAD type: Peripherally Inserted Central Catheter (PICC) | 3.78 [1.28-11.19] * | 0.02 |
| | | | | Body Mass Index (BMI) ≥25 kg/m² | 3.60 [1.31-9.85] * | 0.01 |
| Osamah Al-Asadi 2019 (Osamah Al-Asadi et al., 2019) | All patients with solid cancers who received chemotherapy through the PICC line | 158 | ultrasound Doppler;CT | Smoking | 3.90 [1.09–13.98] | 0.036 |
| | | | | BMI≥ 30 | 1.11 [1.01–1.23] | 0.026 |

PICC = Peripherally Inserted Central Catheter; BMI = Body Mass Index; US = Ultrasound; CVAD = Central Venous Access Device; SPSS = Statistical Package for the Social Sciences; CT = Computed Tomography; * = HR; OR = Odds Ratio; HR = Hazard Ratio; CI = Confidence Interval

Supplementary Table S1 continue.Multivariate analysis: characteristics of included studies

| Author &year | Study population | Sample size | Diagnostic Tools/Diagnostic Methods | Factors | OR/HR [95%CI] | p |
|---|---|---|---|---|---|---|
| Xiao-xia Qiu 2014 (Xiao-xia Qiu et al., 2014) | cancer patients. | 510 | Ultrasonography venography | Vigorous coughing | 6.14 [1.70–22.16] | 0.01 |
| | | | | Severe vomiting | 3.70 [1.28–10.68] | 0.02 |
| Yue Feng 2021 (Yue Feng et al., 2021) | patients with lung cancer | 237 | Color Doppler ultrasonography | Caprini thrombosis risk score | 1.698 [1.103-2.614] | 0.016 |
| | | | | Fibrinogen level | 1.194 [1.004-1.420] | 0.045 |
| | | | | Chemotherapy regimen: Paclitaxel + Cisplatin (vs Pemetrexed + Cisplatin) | 18.332 [2.890-116.278] | 0.002 |
| | | | | Chemotherapy regimen: Gemcitabine + Cisplatin (vs Pemetrexed + Cisplatin) | 6.617 [1.210-36.180] | 0.029 |

OR = Odds Ratio; HR = Hazard Ratio; CI = Confidence Interval

Supplementary Table S1 continue.Multivariate analysis: characteristics of included studies

| Author &year | Study population | Sample size | Diagnostic Tools/Diagnostic Methods | Factors | OR/HR [95%CI] | p |
|---|---|---|---|---|---|---|
| Melina Verso 2008 (Melina Verso et al., 2008) | all randomized patients who received at least one dose of study medication and had adequate venography of the CVC limb or had a confirmed symptomatic pulmonary embolism. | 155 | venography | Inadequate position of CVC tip | 4.05 [1.64–10.02] | 0.002 |
| | | | | Left-sided CVC insertion | 2.29 [1.01–5.51] | 0.05 |
| | | | | Chest radiotherapy | 7.01 [1.42–34.66] | 0.017 |
| | all randomized patients who received at least one dose of study medication and had adequate venography of the CVC limb or had a confirmed symptomatic pulmonary embolism. (placebo group) | 155 | | Distant metastases | 9.36 [1.53–57.05] | 0.01 |
| | | | | Inadequate position of CVC tip | 5.67[1.43-22.40] | 0.01 |
| | | | | Left-sided CVC insertion | 6.01[1.47-24.56] | 0.01 |
| | | | | Weekly CVC care with heparin | 0.041[0.16-1.05] | 0.06 |

CVC = Central Venous Catheter; OR = Odds Ratio; HR = Hazard Ratio; CI = Confidence Interval

Supplementary Table S1 continue.Multivariate analysis: characteristics of included studies

| Author &year | Study population | Sample size | Diagnostic Tools/Diagnostic Methods | Factors | OR/HR [95%CI] | p |
|---|---|---|---|---|---|---|
| JAMES R. 1993 (JAMES R. et al., 1993) | Adult cancer patients (with solid tumors) from the West Kentucky | 255 | The presence of thrombosis was confirmed by injection of contrast material through both the catheter and the ipsilateral upper-extremity venous circulation. | Left-subclavian circulation catheter insertion | / | 0.02 |
| | | | | Antecedent aspiration difficulty ("ball-valve effect", BVE) | / | <0.01 |
| | | | | BVE in left-subclavian circulation | / | 0.038 |
| Puel V. 1993 (Puel V. et al., 1993) | Adult cancer patients who received chemotherapy through implanted venous access devices | 379 | Routine postimplantation chest radiographs and intraoperative fluoroscopy | upper left vs upper right | / | 0.0146 |
| | | | | upper left vs lower right | / | 0.00017 |
| | | | | upper left vs lower left | / | 0.0001 |
| Marcello De Cicco 1997 (Marcello De Cicco et al., 1997) | cancer patients. | 127 | Peripheral phlebography | left subclavian vein catheters than in the right ones | / | <0.01 |
| | | | | higher in subclavian than in innominate | / | <0.001 |
| | | | | the left innominate vein was affected more frequently than the right one | / | <0.001 |
| Junren Kang 2015 (Junren Kang et al., 2015) | breast cancer patients | 568 | Ultrasound Venous duplex ultrasonography | Metastasis | 15.00 [2.75–86.47] | 0.002 |
| | | | | PICC malposition | 18.00 [1.82–178.25] | 0.013 |

BVE = Ball-Valve Effect; PICC = Peripherally Inserted Central Catheter; OR = Odds Ratio; HR = Hazard Ratio; CI = Confidence Interval

Supplementary Table S1 continue.Multivariate analysis: characteristics of included studies

| Author &year | Study population | Sample size | Diagnostic Tools/Diagnostic Methods | Factors | OR/HR [95%CI] | p |
|---|---|---|---|---|---|---|
| Guodong Wang 2025 (Guodong Wang et al., 2025) | adults with cancer requiring PICC insertion | 338 | serial ultrasonography | shallower vessel depth<1.0 cm | 0.324[0.120-0.874] | 0.026 |
| | | | | smaller post-insertion vessel diameter | 0.025[0.001-0.550] | 0.019 |
| Hervé Decousus 2018 | Adult patients with solid tumors who underwent implanted port placement | 3032 | ultrasonography,venography or tomodensitometry | Use of the cephalic vein for catheter insertion (vs jugular/subclavian vein) | 2.51 [1.68-3.75] * | <0.0001 |
| | | | | Ongoing treatment with antiplatelet agents at baseline (vs none) | 0.44 [0.21-0.90] * | 0.024 |
| Guilian Chen 2025 (Guilian Chen et al., 2025) | patients with malignant tumors | 785 | Doppler color ultrasound | Model 1 :Catheter type | 3.95[2.03-7.67] | <0.001 |
| | | | | Model 2： Catheter type | 3.95[2.04-7.67] | <0.001 |
| | | | | Model 3： Catheter type | 3.55[2.05-6.16] | <0.001 |
| | | | | Model 4： Catheter type | 3.47[2.02-5.96] | <0.001 |

PICC = Peripherally Inserted Central Catheter； OR = Odds Ratio； HR = Hazard Ratio； CI = Confidence Interval； *=HR

Supplementary Table S1 continue.Multivariate analysis: characteristics of included studies

| Author &year | Study population | Sample size | Diagnostic Tools/Diagnostic Methods | Factors | OR/HR [95%CI] | p |
|---|---|---|---|---|---|---|
| Mohammad Refaei 2016 (Mohammad Refaei et al., 2016) | Adult inpatients with a new diagnosis of acute leukemia (AL) who received peripherally inserted central catheters (PICC) or centrally inserted central venous catheters (CICC) | 663 | Doppler ultrasonography venogram | Catheter type (PICC vs CICC) | 2.5* | <0.0001 |
| | | | | Repeated catheterizations (2–5 insertions vs 1 insertion) | 2.8* | <0.0001 |
| | | | | Repeated catheterizations (>5 insertions vs 1 insertion) | 3.3* | 0.001 |
| Noailly Charny 2018 (Noailly Charny et al., 2018) | Children with leukemia | 192 | ultrasound | Catheter type: Peripherally Inserted Central Catheter (PICC) | 5.6 [1.2-26.5] | <0.05 |

PICC = Peripherally Inserted Central Catheter; * = HR; OR = Odds Ratio; HR = Hazard Ratio; CI = Confidence Interval; AL = Acute Leukemia; PICC = Peripherally Inserted Central Catheter; CICC = Centrally Inserted Central Venous Catheter

Supplementary Table S1 continue.Multivariate analysis: characteristics of included studies

| Author &year | Study population | Sample size | Diagnostic Tools/Diagnostic Methods | Factors | OR/HR [95%CI] | p |
|---|---|---|---|---|---|---|
| Vineet Chopra 2014 (Vineet Chopra et al., 2014) | Adults who underwent PICC placement | 966 | B-Mode Doppler ultrasound | 5-Fr | 2.21 (1.04–4.70)* | 0.04 |
| | | | | 6-Fr | 3.56 (1.31–9.66)* | 0.01 |
| | | | | PICC gauge | 2.21 [1.04-4.70] * | / |
| Daniel H. Ahn 2013 (Daniel H. Ahn et al., 2013) | cancer who received PICCs at the Dallas Veterans Affairs Medical Center from 2006 to 2009 | 237 | Doppler ultrasound Chest x-ray Blood culture Laboratory tests | use of erythropoiesis-stimulating agents (ESAs) | 10.66[2.25-50.49] | 0.003 |
| | | | | antiplatelet agents (APAs) | 0.25[0.07-0.92] | 0.04 |
| | | | | hospitalization | 2.38[1.05-5.39] | 0.04 |
| | | | | treatment dose anticoagulation (TDA) | 8.34[2.98-23.33] | <0.001 |
| | | | | PICC infection (PI) | 2.46[1.03-5.86] | 0.04 |
| Zeyin Hu 2025 (Zeyin Hu et al., 2025) | patients with cancer receiving PICC insertions | 275 | Doppler sonography | Diabetes requiring insulin | 8.016[1.157–55.536] | 0.035 |
| | | | | Reduced activities of PICC arm | 6.687[2.024–22.090] | 0.002 |
| | | | | Major surgery (> 45 min) | 6.315[1.296–30.77] | 0.023 |

PICC = Peripherally Inserted Central Catheter; Fr = French; ESAs = Erythropoiesis-Stimulating Agents; APAs = Antiplatelet Agents; TDA = Treatment Dose Anticoagulation; PI = PICC Infection; * = HR; OR = Odds Ratio; HR = Hazard Ratio; CI = Confidence Interval

Supplementary Table S1 continue.Multivariate analysis: characteristics of included studies

| Author &year | Study population | Sample size | Diagnostic Tools/Diagnostic Methods | Factors | | OR/HR [95%CI] | p |
|---|---|---|---|---|---|---|---|
| Xuexing Wang 2024 (Xuexing Wang et al., 2024) | cancer patients | 4691 | ultrasound examination | TNM stage | III | 1.706[1.021-2.849] | 0.041 |
| | | | | | IV | 2.177[1.353-3.503] | <0.001 |
| | | | | Activity amount-Frequently | | 0.084[0.043-0.166] | <0.001 |
| | | | | Operation history（yes） | | 1.468[1.058-2.037] | 0.022 |
| | | | | Co-infection（yes） | | 1.796[1.219-2.647] | 0.003 |
| | | | | Hormone use（yes） | | 2.47[1.755-3.476] | <0.001 |
| | | | | Hyperlipidemia（yes） | | 2.448[1.717-3.493] | <0.001 |
| | | | | Thrombosis/Hypercoagulability history（yes） | | 20.831[13.529-32.074 ] | <0.001 |
| | | | | D-Dimer | 0.56-1.00 | 2.687[1.337-5.398] | 0.005 |
| | | | | | 1.01-2.00 | 4.63[2.389-8.972] | <0.001 |
| | | | | | 3.01-4.00 | 10.824[4.763-24.599] | <0.001 |
| | | | | | >4.00 | 8.835[4.466-17.479] | <0.001 |
| Guizhen Weng 2025 (Guizhen Weng et al., 2025) | All patients with solid tumors who received chemotherapy via a PICC line | 605 | Ultrasonography | Anticoagulant therapy (yes) | | 7.549 [2.9-19.652] | <0.05 |

TNM = Tumor-Node-Metastasis； OR = Odds Ratio； HR = Hazard Ratio； CI = Confidence Interval
